# Evolution of Oxygen–Ion and Proton Conductivity in Ca-Doped Ln_2_Zr_2_O_7_ (Ln = Sm, Gd), Located Near Pyrochlore–Fluorite Phase Boundary

**DOI:** 10.3390/ma12152452

**Published:** 2019-08-01

**Authors:** A.V. Shlyakhtina, J.C.C. Abrantes, E. Gomes, N.V. Lyskov, E.Yu. Konysheva, S.A. Chernyak, E.P. Kharitonova, O.K. Karyagina, I.V. Kolbanev, L.G. Shcherbakova

**Affiliations:** 1 N.N. Semenov Federal Research Center for Chemical Physics, Russian Academy of Sciences, ul. Kosygina 4, Moscow 119991, Russia; 2 proMetheus, ESTG, Instituto Politécnico de Viana do Castelo, 4900-348 Viana do Castelo, Portugal; 3 Institute of Problems of Chemical Physics RAS, Moscow region, Chernogolovka 142432, Russia; 4 University of Nottingham Ningbo China, Ningbo 315100, China; 5 Institute of Solid State Chemistry, the Ural Branch of the Russian Academy of Sciences, Pervomayskaya Str. 91, Ekaterinburg 620990, Russia; 6 Moscow State University, Leninskie gory 1, Moscow 119991, Russia; 7 Emanuel Institute of Biochemical Physics, Russian Academy of Sciences, ul. Kosygina 4, Moscow 119991, Russia

**Keywords:** pyrochlore, pyrochlore–fluorite morphotropic phase region, proton conductivity, oxygen—ion conductivity, grain-boundary conductivity, thermogravimetry, Rietveld refinement

## Abstract

Sm_2−x_Ca_x_Zr_2_O_7−x/2_ (x = 0, 0.05, 0.1) and Gd_2−x_Ca_x_Zr_2_O_7−x/2_ (x = 0.05, 0.1) mixed oxides in a pyrochlore–fluorite morphotropic phase region were prepared via the mechanical activation of oxide mixtures, followed by annealing at 1600 °C. The structure of the solid solutions was studied by X-ray diffraction and refined by the Rietveld method, water content was determined by thermogravimetry (TG), their bulk and grain-boundary conductivity was determined by impedance spectroscopy in dry and wet air (100–900 °C), and their total conductivity was measured as a function of oxygen partial pressure in the temperature range: 700–950 °C. The Sm_2−x_Ca_x_Zr_2_O_7−x/2_ (x = 0.05, 0.1) pyrochlore solid solutions, lying near the morphotropic phase boundary, have proton conductivity contribution both in the grain bulk and on grain boundaries below 600 °C, and pure oxygen–ion conductivity above 700 °C. The 500 °C proton conductivity contribution of Sm_2−x_Ca_x_Zr_2_O_7−x/2_ (x = 0.05, 0.1) is ~ 1 × 10^−4^ S/cm. The fluorite-like Gd_2−x_Ca_x_Zr_2_O_7−x/2_ (x = 0.1) solid solution has oxygen-ion bulk conductivity in entire temperature range studied, whereas proton transport contributes to its grain-boundary conductivity below 700 °C. As a result, of the morphotropic phase transition from pyrochlore Sm_2−x_Ca_x_Zr_2_O_7−x/2_ (x = 0.05, 0.1) to fluorite-like Gd_2−x_Ca_x_Zr_2_O_7−x/2_ (x = 0.05, 0.1), the bulk proton conductivity disappears and oxygen-ion conductivity decreases. The loss of bulk proton conductivity of Gd_2−x_Ca_x_Zr_2_O_7−x/2_ (x = 0.05, 0.1) can be associated with the fluorite structure formation. It is important to note that the degree of Ca substitution in such solid solutions (Ln_2−x_Ca_x_)Zr_2_O_7−δ_ (Ln = Sm, Gd) is low, x < 0.1. In both series, grain-boundary conductivity usually exceeds bulk conductivity. The high grain-boundary proton conductivity of Ln_2−x_Ca_x_Zr_2_O_7−x/2_ (Ln = Sm, Gd; x = 0.1) is attributable to the formation of an intergranular CaZrO_3_-based cubic perovskite phase doped with Sm or Gd in Zr sublattice.

## 1. Introduction

An extremely important subject of alternative energy is the development of materials for proton-conducting solid oxide fuel cells (PC-SOFCs). A fuel cell is an electrochemical energy converter, which converts the chemical energy of a fuel (H_2_, CH_4_) and an oxidant (air) to electrical energy. PC-SOFCs are converters, which can use hydrogen as fuel usually at T~ 600–800 °C. In a PC-SOFC the typical electrolyte is ceramic acceptor doped perovskite materials–BaCeO_3_ (BaCe_0.9_Y_0.1_O_3−δ_) and BaZrO_3_ (BaZr_0.8_Y_0.2_O_3−δ_). Acceptor doped BaCeO_3_ has low stability in CO_2_ atmosphere. The main problem of BaZrO_3_-based perovskite materials is low grain-boundary conductivity [1]. Therefore, an important task is to find alternative materials, which have higher or similar proton conductivity compared with well-known BaZr_0.8_Y_0.2_O_3−δ_ perovskite proton conductor, but the problem of low grain-boundary conductivity, which limits the total conductivity of BaZr_0.8_Y_0.2_O_3−δ_, should disappear in these new materials. Although there are numerous experimental studies discussing the effect of chemistry and/or disorder on ionic conductivity of pyrochlores [2,3,4,5,6,7,8,9,10,11,12,13,14], there are only a few that examine the impact of GBs (grain boundaries) on oxygen and proton diffusion in pyrochlores [15,16,17,18].

Ln_2_Zr_2_O_7_ zirconates have long been studied as potential solid electrolytes for SOFCs [2,6,8,9,11,12,13]. Among undoped Ln_2_Zr_2_O_7_ zirconates, the highest oxygen-ion conductivity is offered by Gd_2_Zr_2_O_7_, the most disordered pyrochlore oxide and an intrinsic oxygen-ion conductor (cation anti-site and related oxygen vacancies formation in the pyrochlore structure) [9]. Its oxygen-ion conductivity at 600 °C has been variously reported to be from 3 × 10^−4^ to 7 × 10^−4^ S/cm [2,6,8,9]. Tb_2_Zr_2_O_7_, its neighbor in the lanthanide zirconate series, has the fluorite structure and an order of magnitude lower oxygen-ion conductivity [19,20]. The ordered pyrochlore phase La_2_Zr_2_O_7_ possesses proton conductivity, but it is as low as ~5 × 10^−6^ S/cm at 600 °C [21] (9 × 10^−5^ S/cm at 900 °C [22]). Therefore, proton conductivity is also possible in undoped pyrochlores due to the interaction of intrinsic oxygen vacancies with H_2_O, but since the number of oxygen vacancies is insignificant in the ordered La_2_Zr_2_O_7_, the proton conductivity is also low.

Ca- and Sr-doped light-lanthanide zirconates have oxygen-ion conductivity in dry atmosphere and proton conductivity in wet atmosphere [21,22,23,24,25]. Proton conduction was reliably demonstrated in Ca- and Sr-doped pyrochlore La_2_Zr_2_O_7_ [22,23,24,25]. La_1.95_Ca_0.05_Zr_2_O_6.95_ and La_1.9_Ca_0.1_Zr_2_O_6.9_ pyrochlore solid solutions were reported to be essentially identical in proton conductivity [23,24]: 7 × 10^−4^ S/cm at 600 °C. Obviously, when La_2_Zr_2_O_7_ is doped by calcium, its proton conductivity increases by two orders of magnitude, which is associated with the appearance of extrinsic oxygen vacancies that actively interact with H_2_O in the presence of such a hydrophilic dopant as calcium [25]. The 600 °C proton conductivity of the Sr-doped pyrochlore zirconate La_1.95_Sr_0.05_Zr_2_O_6.95_ is an order of magnitude lower (8 × 10^−5^ S/cm at 600°С) [18]. La_1.95_Sr_0.05_Zr_2_O_6.95_ was annealed in a wide temperature range, including 1600, 1700, and 1900 °C [18]. If the annealing temperature did not exceed 1600 °C, the total conductivity was studied (σ_prot_ = 8 × 10^−5^ S/cm at 600 °C). Annealing at 1700 °C allowed the bulk and grain-boundary components of conductivity to be separately assessed, and the bulk conductivity was found to exceed the grain-boundary component by more than one order of magnitude, reaching 1 × 10^−3^ S/cm at 600 °C. Annealing at the highest temperature, 1900 °C, slightly reduced the bulk conductivity due to deviations from the Sr stoichiometry in the grain bulk, whereas the grain-boundary conductivity increased by half an order of magnitude [18]. Therefore, the rise in grain-boundary conductivity is attributed to the formation of a Sr-containing intergranular phase. In spite of the high bulk proton conductivity, the grain-boundary component limits the total proton conductivity, which is lower (5 × 10^−4^ S/cm at 600 °C) than that of Ca-doped La_2_Zr_2_O_7_ [23,24]. Reducing the Ln ionic radius in the Ln_2−x_M_x_Zr_2_O_7−x/2_ (Ln = La − Lu; M = Ca, Sr) doped zirconate series also leads to a pyrochlore–fluorite morphotropic phase transition. As mentioned above, the intrinsic oxygen-ion conductivity of Ln_2_Zr_2_O_7_ (Ln = La – Gd) increases in going from Ln = La to Gd, so that Gd_2_Zr_2_O_7_ is the most disordered pyrochlore zirconate with the highest oxygen-ion conductivity in this series. It is probably for this reason that Ca doping of Gd_2_Zr_2_O_7_ was unsuccessful, and Fournier et al. [26] observed a decrease in oxygen-ion conductivity with increasing *x* in the Gd_2−x_Ca_x_Zr_2_O_7−x/2_ (x = 0, 0.05, 0.2, 0.3) zirconates synthesized in the range of 1600–1700 °C.

It is important to emphasize that at synthesis temperatures between 1400 and 1900 °C, Moriga et al. [27] observed disordering of the pyrochlore structure of undoped Gd_2_Zr_2_O_7_, whereas synthesis at higher temperatures, above ~1900 °C, yielded fluorite Gd_2_Zr_2_O_7_. It is probably for this reason that Kutty et al. [28], who synthesized Gd_2−x_Sr_x_Zr_2_O_7−x/2_ solid solutions at 1400 °C (within the stability range of ordered pyrochlore Gd_2_Zr_2_O_7_ [27]), found that the oxygen-ion conductivity of Gd_1.9_Sr_0.1_Zr_2_O_6.9_ was twice that of undoped pyrochlore Gd_2_Zr_2_O_7_.

Xia et al. [29] prepared Sm_2−x_Ca_x_Zr_2_O_7−x/2_ (0 ≤ x ≤ 0.1) ceramics at 1700 °C, 10 h and investigated it by impedance spectroscopy in the narrow temperature range of 300–600 °C in air. They observed that electrical conductivity of Sm_2−x_Ca_x_Zr_2_O_7−x/2_ decreases with increasing CaO content. Eurenius et al. [30] measured the proton conductivity of a Sm_2_Zr_2_O_7_-based solid solution, Sm_1.92_Ca_0.08_Zr_2_O_7−x/2_, but they used low-density samples (~70–82%). According to their results, the solid solution has proton conductivity only below 400 °C, and its contribution to the total conductivity is rather small. This differs from data obtained by Shimura et al. [31], who reported the 600 °C conductivity of the Sm_2_Zr_2_O_7_-based solid solution Sm_2_Zr_1.8_Y_0.2_O_7−α_ in hydrogen to be 1 × 10^−4^ S/cm. To the best of our knowledge, the intermediate and heavy lanthanide zirconates have no proton conductivity. Data on the proton conductivity of Gd_2_Zr_2_O_7_-based solid solutions are not available in the literature.

Recently, a Ca-doped 3+/5+ pyrochlore series, which also has a pyrochlore–fluorite morphotropic phase boundary, was shown to have proton conductivity [32], which increases in going from La_2−x_Ca_x_ScNbO_7−x/2_ to Sm_2−x_Ca_x_ScNbO_7−x/2_, i.e., with increasing disorder in the pyrochlore structure, and completely disappears in fluorite Ln_2−x_Ca_x_ScNbO_7−x/2_ (Ln = Ho, Yb).

The purpose of this work is to assess the ratio of oxygen-ion conductivity to proton conductivity in undoped and Ca-doped Sm_2_Zr_2_O_7_ and Gd_2_Zr_2_O_7_ pyrochlores, the compositions of which lie at the pyrochlore–fluorite morphotropic phase boundary. It is of interest to examine how bulk and grain-boundary oxygen-ion conductivity varies in going from the pyrochlores to fluorites and the proton conductivity of the rare-earth zirconate solid solutions gradually disappears. We studied Sm_2−x_Ca_x_Zr_2_O_7−x/2_ (x = 0, 0.05, 0.1) and Gd_2−x_Ca_x_Zr_2_O_7−x/2_ (x = 0.05, 0.1) solid solutions. Note that, to obtain high-density samples, we used the mechanical activation of starting oxides, followed by high-temperature synthesis at 1600 °C. As a result, we obtained disordered Gd_2_Zr_2_O_7_-based solid solutions, more similar in structure to fluorite, whereas the Sm_2_Zr_2_O_7_-based solid solutions had the pyrochlore structure.

## 2. Experimental Part

Sm_2−x_Ca_x_Zr_2_O_7−x/2_ (x = 0, 0.05, 0.1) и Gd_2−x_Ca_x_Zr_2_O_7−x/2_ (x = 0.05, 0.1) were synthesized by reacting appropriate oxide mixtures (Ln_2_O_3_ (Ln = Sm, Gd) + ZrO_2_ + CaO) after mechanical activation in a SPEX8000 ball mill (Glen Mills Inc, Clifton, NJ, USA). The parameters of SPEX8000 mill are: frequency 30 Hz, powder mass −10 g, balls mass −120 g. The Ln_2_O_3_ (Ln = Sm, Gd) starting powders were annealed at 1000 °C for 2 h and then placed in a desiccator after cooling to 850 °C. After the milling, the mixtures were pressed at 914 MPa and then fired at 1600 °C for 4–10 h in air. Gd_1.9_Mg_0.1_Zr_2_O_7−x/2_ was synthesized in the same way, but using MgO as a dopant.

The density of the resultant samples was determined by measuring their mass and dimensions and ranged from 89 to 92.6% of their X-ray density. Characteristics of the compounds and solid solutions under investigation presented in Table 1. All of the synthesized solid solutions were characterized by X-ray diffraction (XRD) on a DRON-3M (Bourevestnik, Sankt-Petersburg, Russia) (filtered Cuk_α_ radiation, step scan mode with a step of 0.1 or 0.05°, angular range 2θ = 10–65°). In addition, for the same synthesized solid solutions the XRD patterns were registered on a Bruker D8 Advance diffractometer (Bruker AXS, Karlsruhe, Germany) in the reflection mode with Ni-filtered CuKα radiation. The diffractometer is equipped with a LynxEYE detector. The XRD patterns were registered under air at the temperature of 22 °C in the angular range of 12 ≤ 2θ ≤ 98 with a step size of 0.01° and counting for 0.3 s in each point. Corundum (Bruker AXS, Karlsruhe, Germany) and Si powder (Sigma-Aldrich, St. Louis, MI, USA) were used as the external and internal standards.

The microstructure of the sintered ceramics was examined using scanning electron microscopy (JEOL JSM-6390LA, JEOL, Tokyo, Japan).

Thermogravimetric analysis was performed by using the NETZSCH STA 449C system (Netzsch, Selb, Germany) (30–1000 °C, heating rate of 10 K/min, Al_2_O_3_ plate) in air. A detailed description of the experiment can be found in Reference [32].

For electrical measurements disk-shaped polycrystalline samples (diameter **~**9 mm and thickness 2–3 mm) were prepared. Contacts to the sample faces were made by firing ChemPur C3605 paste, containing colloidal platinum, at 950–1000 °C. The conductivity of Sm_2−x_Ca_x_Zr_2_O_7−x/2_ (x = 0, 0.05, 0.1) и Gd_2−x_Ca_x_Zr_2_O_7−x/2_ (x = 0.05, 0.1) was characterized by impedance spectroscopy in dry and wet air. Electrical conductivity measurements of the samples were performed on cooling regime using a P-5X potentiostat/galvanostat combined with frequency response analyzer module (Elins Ltd., Russia) over the frequency range of 0.1 Hz to 500 kHz at signal amplitude of 150 mV in the temperature range of 100–900 °C. Dry atmosphere was created by passing air through a KOH and wet atmosphere through a water saturator held at 20 °C, which ensured constant humidity of about 0.023 atm (2.3% H_2_O). Air flow rate was 130 mL/min. To get stable state (water vapor pressure) before conductivity measurement, the sample was kept at each temperature for 40 min. The impedance data fitting was performed by the least squares refinement program ZView (Scribner Associates Inc., Southern Pines, NC, USA). The general equivalent circuit model used to fit the experimental data includes at least two (RQ)-circuits connected in series, where R is the resistance and Q is the constant phase element. Most of the spectra consist of high- and low-frequency arcs. The high-frequency (from 500 to ~0.1–1 kHz) arc corresponds to the bulk (R_b_) and grain-boundary (R_gb_) resistances of the sample, and the low-frequency (from ~0.1–1 kHz to 1 Hz) arc represents the electrode polarization resistance.

Electrical characterization of Sm_2−x_Ca_x_Zr_2_O_7−x/2_ (x = 0.05, 0.1) and Gd_2−x_Ca_x_Zr_2_O_7−x/2_ (x = 0.1) was carried out by impedance spectroscopy in the frequency range of 20 Hz to 1 MHz, with a signal amplitude of 200 mV, using a Hewlett-Packard 4284A precision LCR bridge, as a function of the oxygen partial pressure, during reoxidation, after reduction with a mixture of 95% N_2_ and 5% H_2_, between 700 and 950 °C.

## 3. Results and Discussion

### 3.1. Structure of Sm_2−x_Ca_x_Zr_2_O_7−x/2_ (x = 0, 0.05, 0.1) and Gd_2−x_Ca_x_Zr_2_O_7- x/2_ (x = 0.05, 0.1) Studied by XRD with Rietveld Refinement

**Figure 1:**
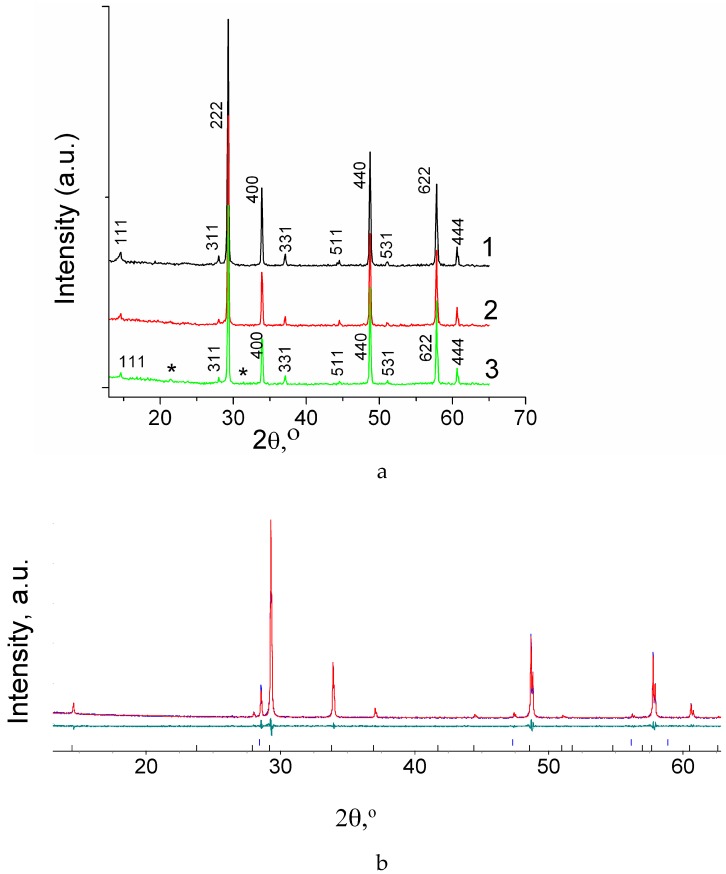
(**а**) XRD patterns of the Sm_2−x_Ca_x_Zr_2_O_7−x/2_(1) x = 0, (2) x = 0.05, (3) x = 0.1; (**b**) Rietveld data of Sm_2−x_Ca_x_Zr_2_O_7−x/2_ (x = 0.05) XRD pattern: the measured (blue line), the calculated (red line), the difference between measured and calculated data (green line). Vertical bars show calculated reflections for different phases Sm _1.95_Ca_0.05_Zr_2_O_6.975_ (lower) and Si internal standard (upper). *R*_wp_ = 3.36%, *R*_p_ = 4.49%, *R*_exp_ = 3.47%, GOF = 1.34.

**Figure 2:**
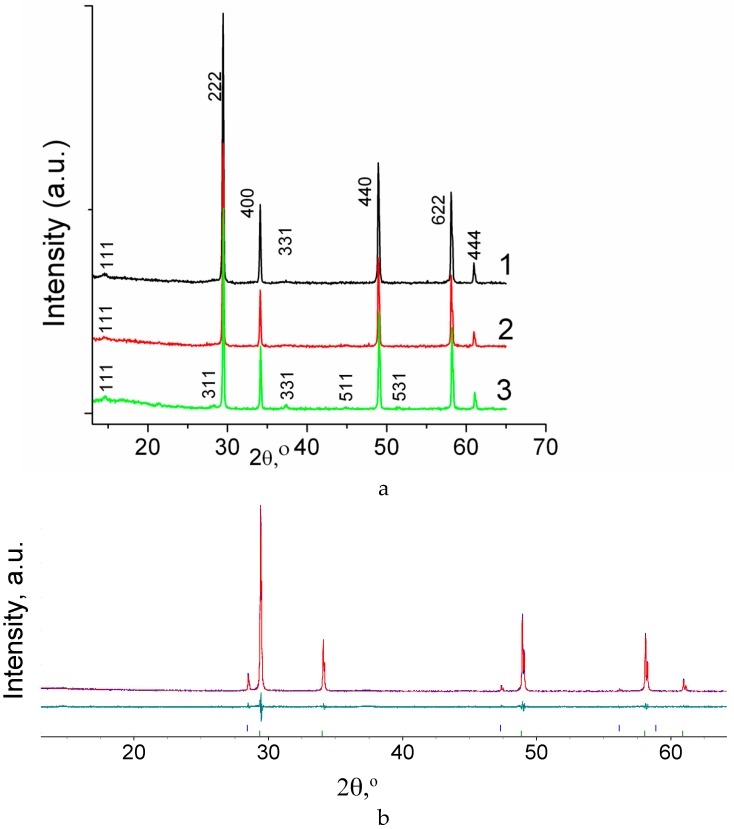
(**a**) XRD patterns of the Gd_2−x_Ca_x_Zr_2_O_7−x/2_ (1) x = 0.05, (2) x = 0.1, and (3) Gd_1.9_Mg_0.1_Zr_2_O_6.95_; (**b**) Rietveld data of Gd_2−x_Ca_x_Zr_2_O_7−x/2_ (x = 0.05) XRD pattern: the measured (blue line), the calculated (red line), the difference between measured and calculated data (green line). Vertical bars show calculated reflections for different phases Gd_1.95_Ca_0.05_Zr_2_O_6.975_ (lower) and Si internal standard (upper). *R*_wp_ = 2.92%, *R*_p_ = 3.67%, *R*_exp_ = 2.82%, GOF = 1.26.

Figure 1a and Figure 2a present XRD results for the Sm_2−x_Ca_x_Zr_2_O_7−x/2_ (x = 0, 0.05, 0.1) and Gd_2−x_Ca_x_Zr_2_O_7−x/2_ (x = 0.05, 0.1) solid solutions. It is seen that, upon doping with calcium, which has a larger ionic radius than do the lanthanides (R_CN8_ Sm^3+^ = 1.079, R_CN8_ Gd^3+^ = 1.053, R_CN8_ Ca^2+^ = 1.12 Å), all of the Sm-containing materials retain the pyrochlore structure, but the intensity of the pyrochlore superstructure reflections decreases with increasing doping level (Figure 1a). In the case of the Gd_2−x_Ca_x_Zr_2_O_7−x/2_ (x = 0.05, 0.1) solid solutions, synthesized at the same temperature, 1600 °C, there is only one pyrochlore superstructure reflection, (111), and Gd_2−x_Ca_x_Zr_2_O_7−x/2_ (x = 0.05) has a weak (331) line. The Gd_1.9_Mg_0.1_Zr_2_O_6.95_ solid solution, containing Mg, which has a smaller ionic radius than does Gd (R_CN8_ Gd^3+^ = 1.053, R_CN8_ Mg^2+^ = 0.89 Å), has the pyrochlore structure and its XRD pattern shows the (111), (311), (331), (511), and (531) superstructure reflections (Figure 2a, scan 3). Therefore, Ca substitution on the lanthanide site leads to disordering (decreasing and disappearing of (111), (311), (331), (511), and (531) superstructure reflections) in the pyrochlore structure of Ln_2_Zr_2_O_7_ (Ln = Sm, Gd) (Figure 1a and Figure 2a).

The structure of the solid solutions was refined by the Rietveld method. The results are presented in Table 2, Table 3 and Table 4 and, for the *x* = 0.05 solid solutions, in Figure 1b and Figure 2b. It is seen from Table 2 that the lattice parameter of Sm_1.95_Ca_0.05_Zr_2_O_6.975_ (*a* = 10.5925(1) Å) is lower than that of undoped Sm_2_Zr_2_O_7_ (*a* = 10.5975(1) Å). On Ca-doping of Nd_2_Zr_2_O_7_ [33] the change of Ca coordination number from 8 to 7 in the pyrochlore structure was observed by neutron diffraction (ND) at room temperature. Then the ionic radii of the host ions Sm^3+^_CN8_ will be larger than that the ionic radii of dopant Ca_CN7_ (R_CN8_ Sm^3+^ = 1.079 Å, R_CN7_ Ca^2+^ = 1.06 Å) compare to undoped Sm_2_Zr_2_O_7_ (R_CN8_ Sm^3+^ = 1.079 Å, R_CN8_ Ca^2+^ = 1.12 Å). Therefore, it is reasonable to expect the decrease in the lattice parameters of Sm_1.95_Ca_0.05_Zr_2_O_6.975_. The data presented in Table S1 for Sm_1.95_Ca_0.05_Zr_2_O_6.975_ illustrate that there is a small variation in the A- and B- sites occupancies by host and doping cations (at the same quality of refinement) resulting in the appearance of the cation anti-sites generated by host cations and incorporation of Ca cations into both A-sublattice (preferably) and B-sublattice. According to the Rietveld refinement of Sm_1.9_Ca_0.1_Zr_2_O_6.95_ the variation in the A- and B-sites occupancies could occur with a higher degree of the formal substitution Table S2. In the latest case, the fraction of the cation anti-sites generated by the host cations could be higher (up to 8% on both sublattices). The lattice parameter of the Sm_1.9_Ca_0.1_Zr_2_O_6.95_ solid solution (5% substitution), is *а* = 10.5923(1) Å (Table 2), is almost comparable with that for Sm_1.95_Ca_0.05_Zr_2_O_6.975_ suggesting that a solubility limit of calcium in the pyrochlore structure is within 0.05 < x < 0.10. Note the additional lines (100) and (110) at 2θ~22° and 31.5° exist in the XRD pattern of Sm_1.9_Ca_0.1_Zr_2_O_6.95_ (Figure 1a, scan 3; the additional lines are marked by asterisks). These lines correspond to the main diffraction lines of CaZrO_3_ perovskite. In the study of the oxygen-ion conductivity of the Sm_2−x_Ca_x_Zr_2_O_7-δ_ series, the appearance of tiny peaks of perovskite-like CaZrO_3_ second phase was observed in the XRD pattern of Sm_1.9_Ca_0.1_Zr_2_O_6.95_ (x = 0.1) solid solution [29]. With the appearance of the second phase, the real composition of this sample could deviate from the intended composition, nevertheless the Rietveld refinement of the X-ray diffraction data was carried out for Sm_1.9_Ca_0.1_Zr_2_O_6.95_ and corresponding crystallographic information is presented in Table 2 and Table S2 in order to give insight into the defect formation at a higher degree of Ca substitution. Most likely, the second phase appears due to the interaction of excess Ca, which cannot fully enter to the samarium sublattice, with zirconium.

Previously, in studies of Sr-doped Gd_2_Zr_2_O_7_ and Gd_2_Hf_2_O_7_, Gd_1.8_Sr_0.2_Zr_2_O_6.9_ and Gd_1.8_Sr_0.2_Hf_2_O_6.9_ samples (10% substitution) were found to contain SrZrO_3_ and SrHfO_3_ perovskites, respectively, as impurity phases [28,34]. These findings confirm that the degree of Sr substitution for Gd in Gd_2_Zr_2_O_7_ and Gd_2_Hf_2_O_7_ does not exceed 5%. An excess of Sr above 5% forms the SrMO_3_ (M = Zr, Hf) perovskite compounds, and their lines emerge in XRD patterns. In this study, in the case of Sm_1.9_Ca_0.1_Zr_2_O_6.95_ we assume the formation of a small amount perovskite CaZrO_3_ based phase, because the observed extra lines are similar to the (100) and (110) lines of cubic perovskite CaZrO_3_ [35]. Clearly, there will be deviations from the intended stoichiometry in the grain bulk of the Sm_1.9_Ca_0.1_Zr_2_O_6.95_ solid solution.

The Rietveld refinement was carried out for Gd_2−x_Ca_x_Zr_2_O_7−x/2_ (x = 0.05, 0.1) compositions as well (Table 2 and Figure 2b). Two structural models were considered for each composition: the pyrochlore structure and the fluorite structure with oxygen deficiency corresponding to the oxygen content in the pyrochlore structure. The Rietveld refinement carried out for Gd_2−x_Ca_x_Zr_2_O_7−x/2_ (x = 0.05, 0.1) compositions as for a single phase compound with the phyrochlore structure indicates that a wide variation in occupancies of A-sites and B-sites results in the same set of the refinement factors. The following variations in occupancy were observed: Gd_Gd_ ~ 0.48–0.62, Zr_Gd_ ~ 0.36–0.50, Zr_Zr_ ~ 0.50–0.64, Gd_Zr_ ~ 0.35–0.50 for Gd_1.9_Ca_0.05_Zr_2_O_6.975_ as well as Gd_Gd_ ~ 0.46–0.65, Zr_Gd_ ~ 0.30–0.54, Zr_Zr_ ~ 0.46–0.70, Gd_Zr_ ~ 0.30–0.49 for Gd_1.9_Ca_0.1_Zr_2_O_6.95_. The typical sets of the refinement factors are presented in Table 2. Location of Ca cations on the A-site only, B-sites only or on both A- and B-sites in different ratios will not change the quality of the refinement. This indicates a high degree of disorder within the pyrochlore structure. The comparison of the Rietveld refinement for the two structural models indicates that both Gd_2−x_Ca_x_Zr_2_O_7−x/2_ (x = 0.05, 0.1) compositions are better described by the fluorite structural model as the refinement factors are slightly better (Table 2). The lattice parameters of Gd_2−x_Ca_x_Zr_2_O_7−x/2_ (x = 0.05, 0.1) compositions as for compounds with fluorite structure are two times less when these compounds are described by pyrochlore structural model. The description of Gd_2−x_Ca_x_Zr_2_O_7−x/2_ (x = 0.05, 0.1) compositions within the pyrochlore structure model allows us to consider the evolution of lattice parameters on Ca-doping. The lattice parameter of the Gd_1.95_Ca_0.05_Zr_2_O_6.975_ solid solution (2.5% substitution) was determined to be 10.5326(1) Å. Undoped Gd_2_Zr_2_O_7_ was investigated in Reference [35,36,37]. According to previous studies, the lattice parameter of undoped Gd_2_Zr_2_O_7_ is 10.524 Å [36] or 10.528 Å [37]. Therefore, the lattice parameter of the Gd_1.95_Ca_0.05_Zr_2_O_6.975_ solid solution (2.5% Ca substitution) exceeds that of undoped Gd_2_Zr_2_O_7_. In this case, raising the degree of substitution to 5% (Gd_1.9_Ca_0.1_Zr_2_O_6.95_) does not actually change the lattice parameter (*a* = 10.5321(1) Å). In this case, we also assume the presence of a small amount (under 5%) of a second phase, based on perovskite CaZrO_3_. Recently, a series of Gd_2−x_Ca_x_Zr_2_O_7−x/2_ (x = 0, 0.02, 0.05, 0.1, 0.15, 0.2, 0.25, 0.3) solid solutions was synthesized by a hydrothermal process followed by annealing at 1500 °C for 4 h [38]. The lattice parameter of Gd_1.95_Ca_0.05_Zr_2_O_6.975_ (2.5% substitution) was found to exceed that of undoped Gd_2_Zr_2_O_7_. The ionic radii of the host Gd cations (R_CN8_ Gd^3+^= 1.056 Å) is smaller than that the ionic radii of dopant Ca^2+^ (R_CN7_ Ca^2+^ = 1.06 Å). Therefor it is reasonable to expect the increase in the parameter of Gd_1.95_Ca_0.05_Zr_2_O_6.975_ in comparison with Gd_2_Zr_2_O_7_. At higher doping levels, the lattice parameter remained essentially constant up to *x* = 0.15. Starting at 5% substitution (Gd_1.9_Ca_0.1_Zr_2_O_6.95_), diffraction lines of a second phase, perovskite GdZrO_3_, were observed [38]. These data are completely consistent with our results (Table 2).

### 3.2. Microstructure of Sm_2−x_Ca_x_Zr_2_O_7−x/2_ (x = 0, 0.1)

**Figure 3:**
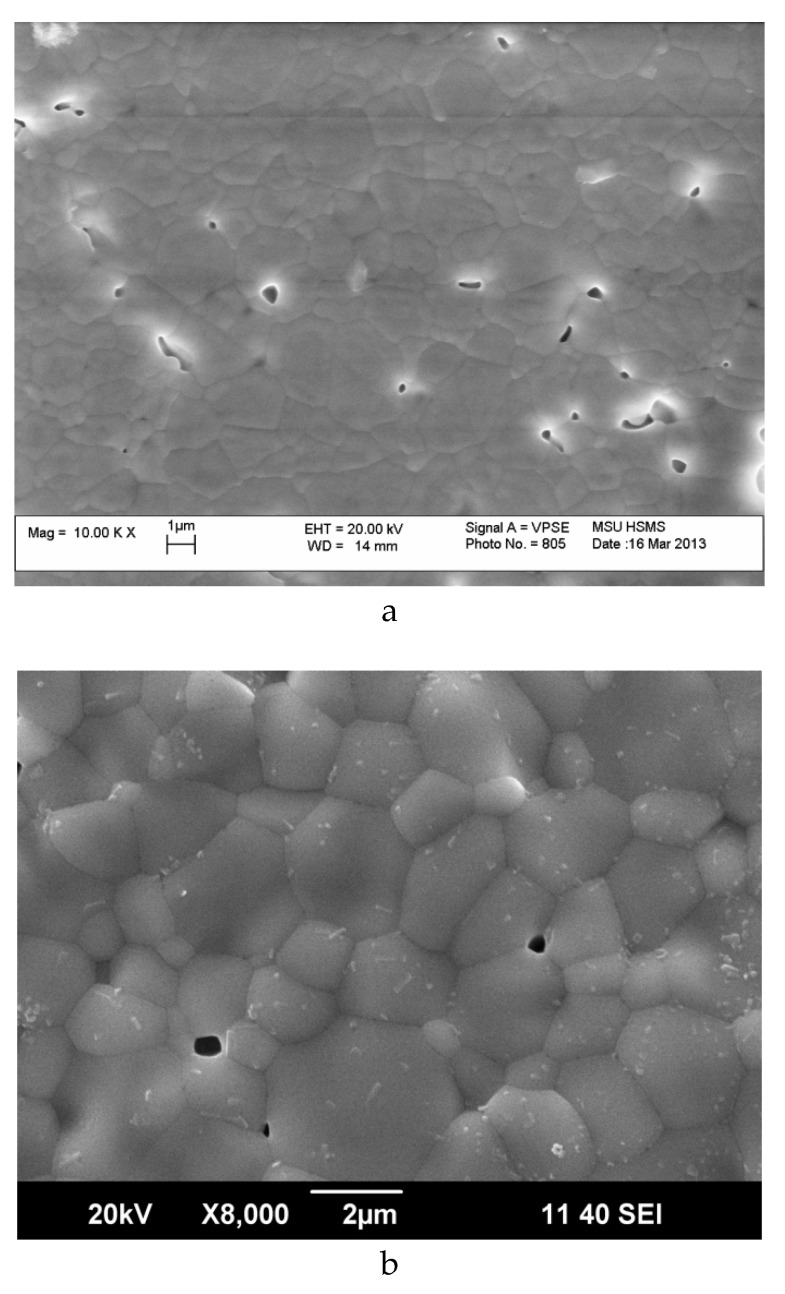

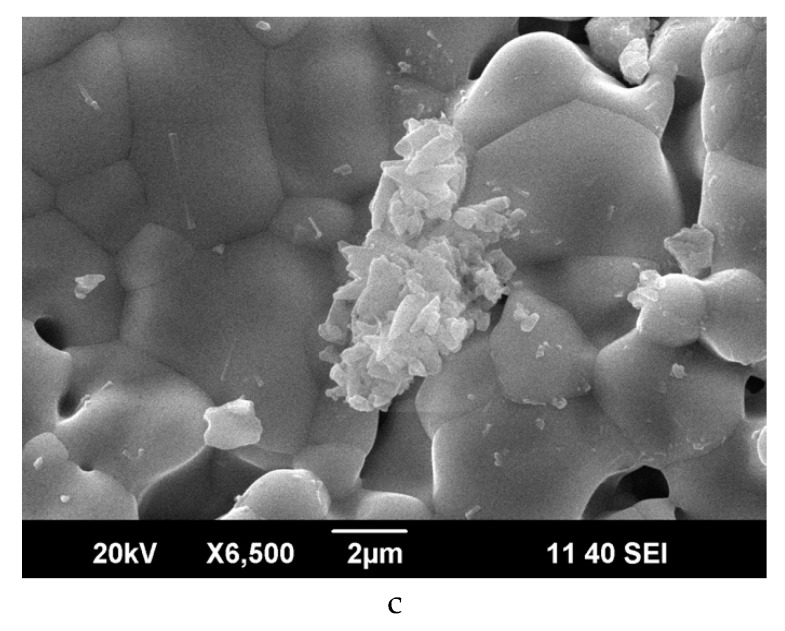
SEM images of (**a**) Sm_2_Zr_2_O_7_ ceramics; (**b**) Sm_1.9_Ca_0.1_Zr_2_O_6.95_ ceramics; (**c**) the micron-size agglomerate formed from small grains in Sm_1.9_Ca_0.1_Zr_2_O_6.95_ ceramics.

Figure 3 illustrates the microstructure of undoped Sm_2_Zr_2_O_7_ and Sm_1.9_Ca_0.1_Zr_2_O_6.95_ ceramics. The open porosity of both ceramics is insignificant. However, in Sm_1.9_Ca_0.1_Zr_2_O_6.95_ ceramics grains with the size of 100–300 nm and micron-size agglomerates formed from small grains can be observed in addition to well-sintered micron-size grains (Figure 3b,c). The small grains (100–300 nm) are randomly distributed over the ceramics. SEM/EDX point analysis was used to identify chemical composition of different microstructural components in Sm_1.9_Ca_0.1_Zr_2_O_6.95_ ceramics and compare with the nominal stoichiometry of this composition (Table 3). Well-sintered micron-size grains contain 1.7–2.5 at.% Ca, which is comparable with the nominal stoichiometry. In contrast to the nominal stoichiometry, the concentration of Sm cations is larger than that of Zr. At the grain boundaries and in the neighboring area, the concentration of Ca (1.6–2 at.%) is comparable with that in the bulk of the grains. A similar trend was revealed for the grains with the size of 100–300 nm on the surface of micron-size well co-sintered grains. It is necessary to note that the analyzing volume, which is defined by instrumentation, is much larger (sub-micron/micron range) than the grain boundaries and grains with the size of 100–300 nm, therefore the measured values have uncertain contributions from the bulk of surrounded grains and cannot describe precisely chemical composition of the grain boundaries and grains with the size of 100–300 nm in the ceramics. Therefore, one cannot rule out the formation around the grains a thin nano-size film with different chemical composition and deviation in Ca content in the grains with the size of 100–300 nm. A slightly higher concentration of Ca (up to 4.9 at%) was revealed in individual small grains with the size of 0.6–1.1 mkm. A higher concentration of Sm was detected for these grains as well, but much higher variation in the absolute values was observed: 49.1–73.6 at.% (Table 3). A high concentration of Ca in micron-size agglomerates formed from small grains is evidently confirmed through the mapping of Ca, Sm and Zr (Supplementary information, Figure S1). The ratio [Ca]/([Sm] + [Zr]) is close to 1–1.5, which allows us to assume the formation of Ca based perovskite containing both Sm and Zr on the B-sites.

### 3.3. TG Characterization of Sm_2−x_Ca_x_Zr_2_O_7- x/2_ (x = 0, 0.1) and Gd_2−x_Ca_x_Zr_2_O_7- x/2_ (x = 0.1)

The geometric densities of the synthesized samples are presented in Table 1. Note that they fall in the range of 89–92.6%, markedly exceeding those reported previously [30].

**Figure 4:**
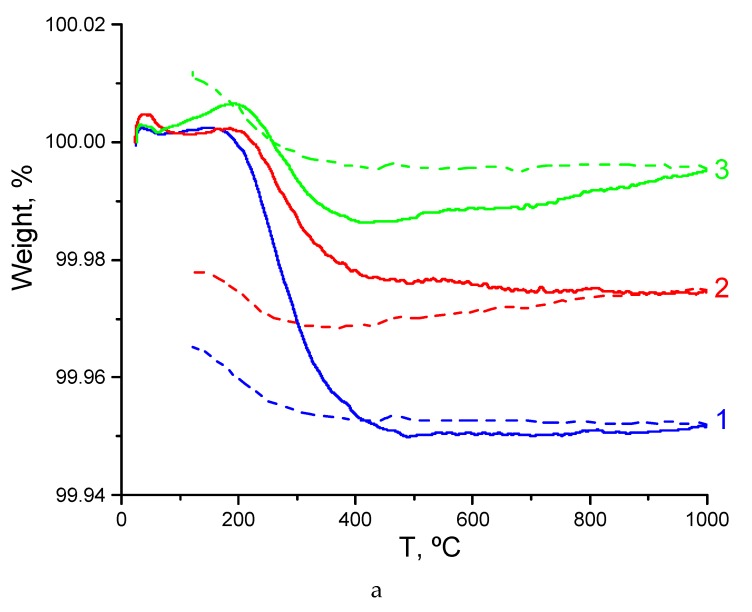

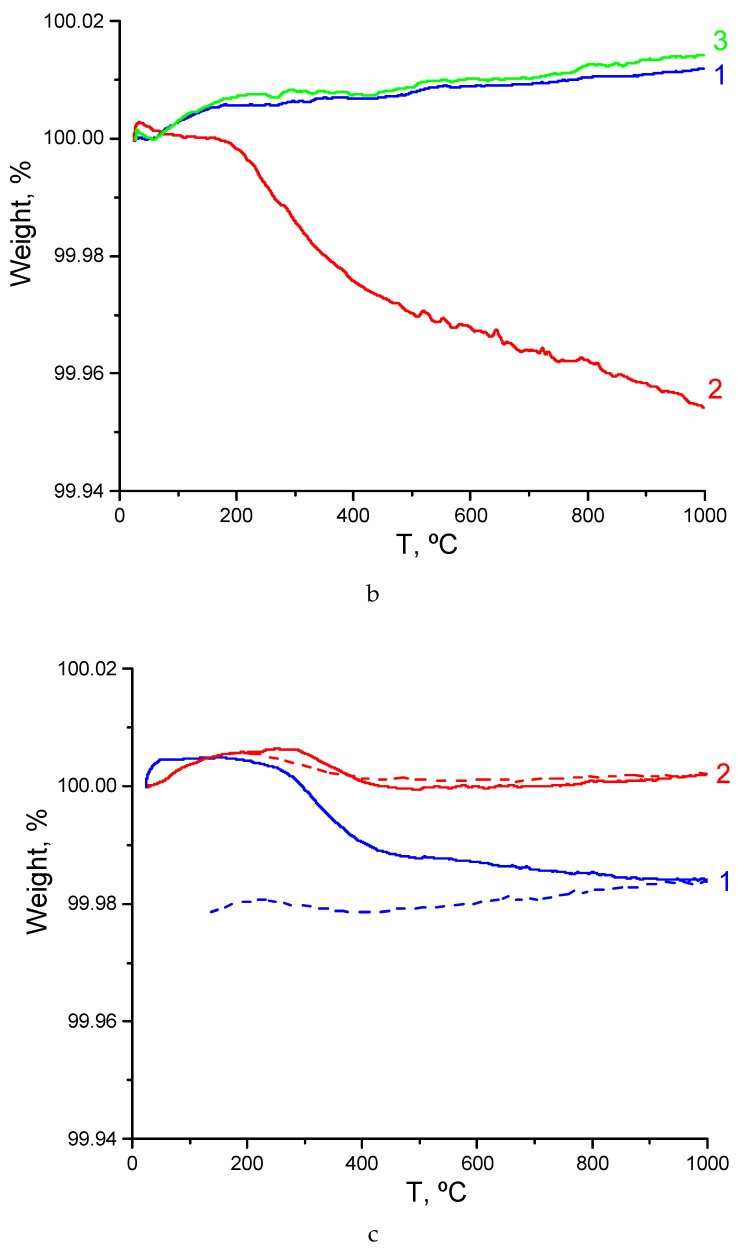
TG curves for (**a**) newly synthesized sample Sm_2_Zr_2_O_7_ (1–1st heating–cooling; 2–2nd heating–cooling; 3–3rd heating–cooling cycles); (**b**) newly synthesized and hydrated for 4 weeks samples Sm_1.9_Ca_0.1_Zr_2_O_6.95_ (1–newly synthesized sample 1st heating; 2–hydrated sample 1st heating; 3–hydrated sample 2nd heating); (**c**) newly synthesized sample Gd_1.9_Ca_0.1_Zr_2_O_6.95_ (1–1st heating–cooling; 2–2nd heating–cooling cycles). The solid line indicates the heating stage, the dashed line indicates the cooling stage.

Figure 4a presents the results obtained for as-synthesized pyrochlore Sm_2_Zr_2_O_7_ in three successive heating–cooling cycles between 25 and 1000 °C in air. It is seen that the initial weight loss is 0.05% and that essentially all of the water is removed below 500 °C, but during cooling the material partially picks up water from air. The same is observed in the next two cycles. Water is readily removed from Sm_2_Zr_2_O_7_ and then partially absorbed during cooling. Sm_2_Zr_2_O_7_ loses water in one step, in the temperature interval of 250–500 °C, like hydrophilic samarium oxide Sm_2_O_3_ [39]. It seems likely that, for the most part, surface water and hydroxyl ions are involved. Prolonged hydration and higher temperatures seem to be needed for structurally bound water and interstitial protons to be present in Sm_2_Zr_2_O_7_. Note that the present TG curves of Sm_2_Zr_2_O_7_ are similar to those obtained by Eurenius et al. [30] for different Ca-doped Sm_2_B_2_O_7-δ_ (B = Ti, Sn, Zr and Ce) proton-conducting pyrochlore oxides.

Figure 4b presents analogous experimental data for pyrochlore Sm_1.9_Ca_0.1_Zr_2_O_6.95_. It is seen that there is no water loss (Figure 4b, curve 1). Next, pyrochlore Sm_1.9_Ca_0.1_Zr_2_O_6.95_ was held in water for four weeks and characterized by TG (Figure 4b, curves 2, 3). The hydrated Sm_1.9_Ca_0.1_Zr_2_O_6.95_ was heated twice to 1000 °C. During the first heating, surface water, the water in the pores of the ceramic, and hydroxyl ions were removed below about 500 °C (~0.03%) [40]. Above 500 °C, water was removed rather slowly, without obvious steps, up to 1000 °C. Clearly, structurally bound water and interstitial protons were involved [40]. The amount of weight loss was about 0.015% at 500–1000 °C. The second heating caused no weight loss. Therefore, the kinetics of water incorporation into the structure of Sm_1.9_Ca_0.1_Zr_2_O_6.95_ is extremely sluggish. Nevertheless, in this sample there is strongly bound water and interstitial protons, as distinct from undoped Sm_2_Zr_2_O_7_, which has a comparable density.

Figure 4c shows TG heating and cooling curves obtained for the Gd_1.9_Ca_0.1_Zr_2_O_6.95_ solid solution without prehydration. Here, during the first heating we observe the first weight loss stage of ~0.02% below 450 °C, which is obviously due to the removal of surface water and hydroxyl ions, and a second weight loss stage of ~0.005% in the range of 450–1000 °C, which is due to the structurally bound water and interstitial protons [40]. Note that the cooling curves of the Gd_1.9_Ca_0.1_Zr_2_O_6.95_ sample have small anomalies in the range of 200–300 °C, due to water sorption from the atmosphere, but they are much smaller than those of the undoped Sm_2_Zr_2_O_7_ sample. Therefore, there is negligible weight loss during the second heating.

We believe that if structurally bound water and interstitial protons do not show up in TG curves between 500 and 1000 °C, this may mean that the samples should be prehydrated, and this is related to kinetic hindrances for water incorporation into the complex defect structure of the mixed oxides. This should be taken into account in proton conductivity measurements, because even prolonged holding, up to 5 h, at each temperature can be insufficient for incorporating water into the defect structure of the mixed oxides, and the results will be underestimated.

### 3.4. Conductivity of the Sm_2−x_Ca_x_Zr_2_O_7- x/2_ (x = 0, 0.05, 0.1) Solid Solutions in Dry and Wet Air

**Figure 5:**
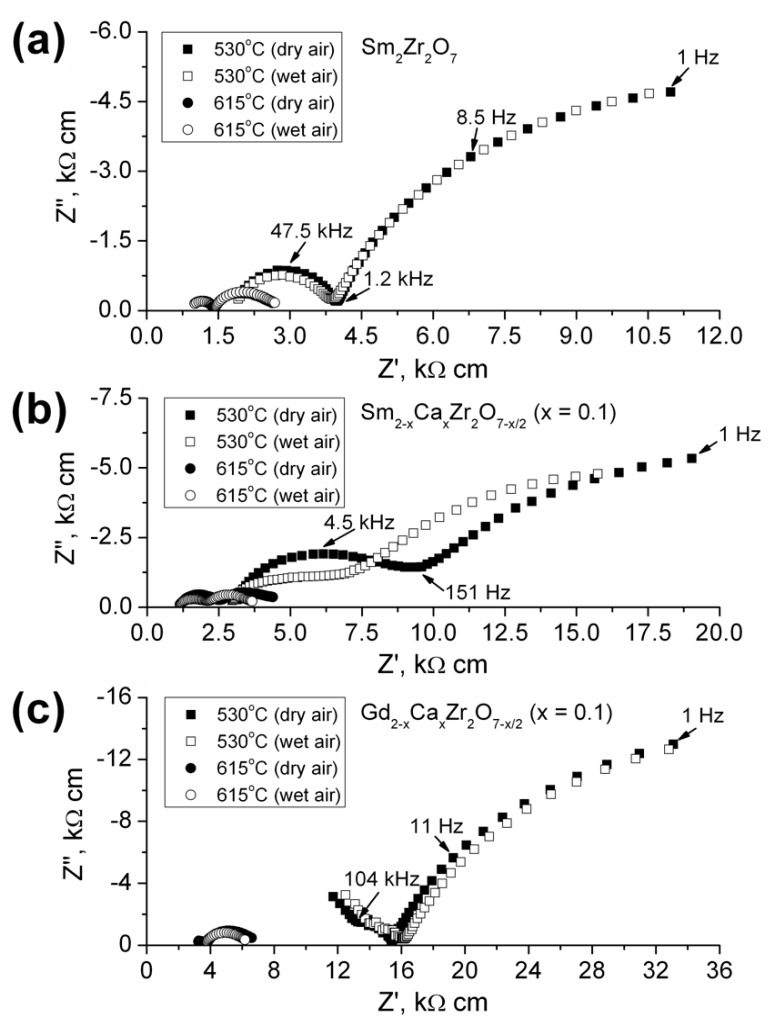
Impedance spectra of (**a**) Sm_2_Zr_2_O_7_, (**b**) Sm_2−x_Ca_x_Zr_2_O_7−x/2_ (x = 0.1), and (**c**) Gd_2−x_Ca_x_Zr_2_O_7−x/2_ (x = 0.1) at 530 and 615 °C in dry and wet air.

Figure 5 shows impedance spectra of undoped Sm_2_Zr_2_O_7_ and the Ln_2−x_Ca_x_Zr_2_O_7−x/2_ (Ln = Sm, Gd; x = 0.1) solid solutions (5% substitution) in dry and wet air at 530 and 615 °C, respectively. Most of the spectra have the form of two arcs at high (0.1–1 to 500 kHz) and low (0.1–1 kHz to 1 Hz) frequencies. The high-frequency arc represents the bulk (*R_b_*) and grain-boundary (*R_gb_*) resistances of the sample, and the low-frequency arc represents the electrode polarization resistance. *R*_b_ and *R*_gb_ were evaluated by extrapolating the high-frequency arc to the real axis: *R*_b_ corresponds to the high-frequency limit (>500 kHz), and *R*_gb_, to intermediate frequencies (0.1–1 kHz). The specific capacitances corresponded to the bulk, grain boundary, and electrode arcs were ~10^−11^ F cm^−1^, ~10^−7^÷10^−8^ F cm^−1^, and ~10^−5^ F cm^−1^, respectively.

**Figure 6:**
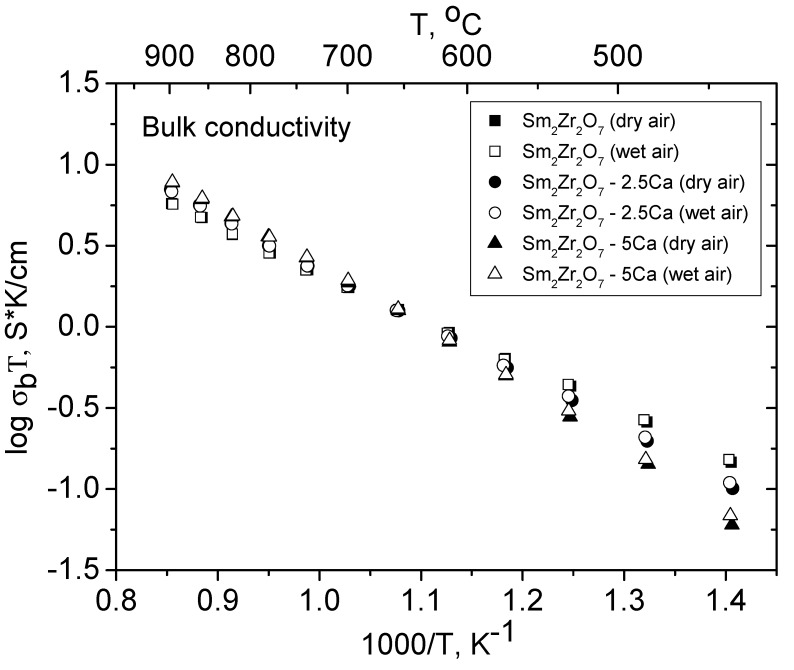
Bulk conductivity of Sm_2−x_Ca_x_Zr_2_O_7−x/2_ (x = 0, 0.05, 0.1) in dry and wet air.

**Figure 7:**
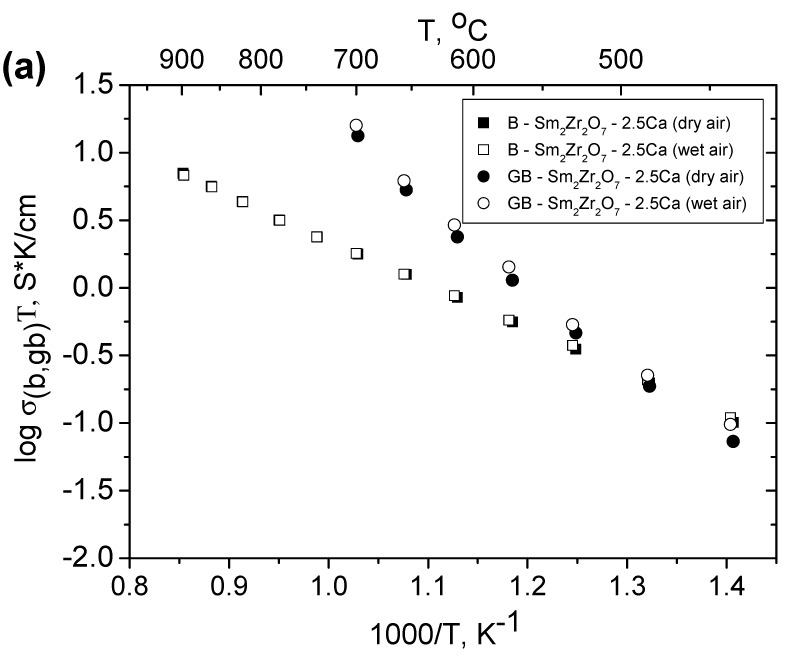

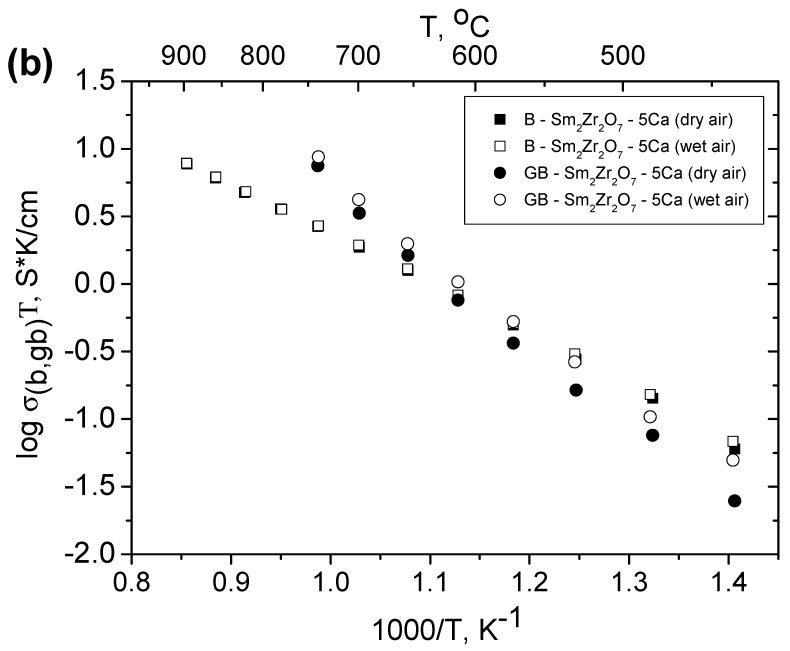
Bulk and grain-boundary conductivities of (**a**) Sm_2−x_Ca_x_Zr_2_O_7−x/2_ (x = 0, 0.05) and (**b**) Sm_2−x_Ca_x_Zr_2_O_7−x/2_ (x = 0, 0.1) in dry and wet air.

**Figure 8:**
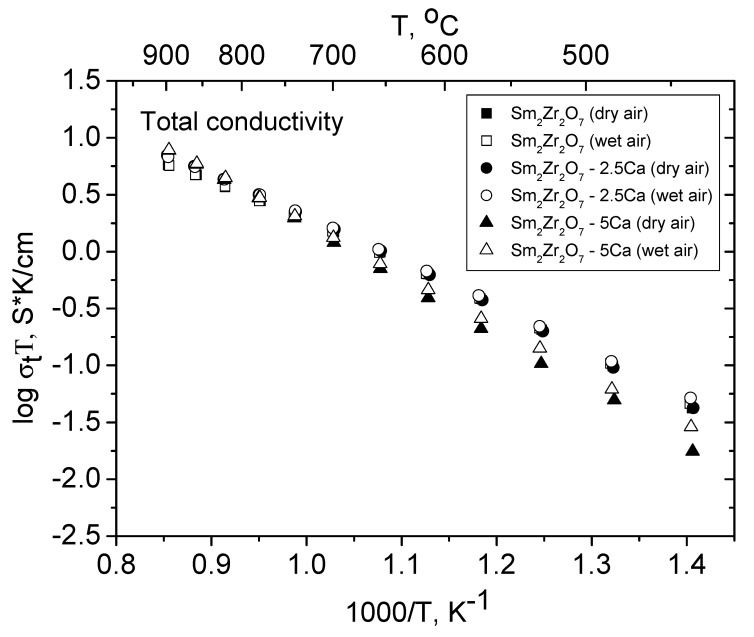
The total conductivity of Sm_2−x_Ca_x_Zr_2_O_7−x/2_ (x = 0, 0.05, 0.1) in dry and wet air.

Figure 6, Figure 7 and Figure 8 and Table 4 summarize the bulk, grain-boundary, and total conductivities of the Sm_2−x_Ca_x_Zr_2_O_7−x/2_ (x = 0, 0.05, 0.1) solid solutions in dry and wet air. Above 700 °C, the Sm_2−x_Ca_x_Zr_2_O_7−x/2_ (x = 0.05, 0.1) solid solutions have the conductivity, which is independent of humidity (Figure 6). According to López-Vergara et al. [41], this means that oxygen-ion conductivity prevails at these temperatures. The activation energies for conduction in dry and wet atmospheres are indicated in Table 4. It is seen that the activation energies for conduction in all of the Sm_2−x_Ca_x_Zr_2_O_7−x/2_ (x = 0, 0.05, 0.1) samples in dry air lie within the range 0.7–0.95 eV, characteristic of similar oxygen-ion-conducting systems [13,42,43,44,45]. Note that there is the Ca-doping effect above 700 °C in the pure oxygen-ion conduction region: the bulk conductivity of the Ca-doped solid solutions exceeds that of undoped Sm_2_Zr_2_O_7_.

It is seen (Table 4) that below 600 °C the activation energies for conduction in Sm_2−x_Ca_x_Zr_2_O_7−x/2_ (x = 0, 0.05, 0.1) samples in wet air is lower than that of dry air. This is typical for proton-conducting oxides.

Below 600 °C, the bulk conductivity of Sm_2_Zr_2_O_7_ exceeds that of the Sm_2−x_Ca_x_Zr_2_O_7−x/2_ (x = 0.05, 0.1) solid solutions (Figure 6). Increasing the degree of substitution reduces the bulk conductivity of the solid solutions. Below 600 °C, protons contribute to conduction in both Sm_2_Zr_2_O_7_ and the Ca-doped solid solutions. However, the influence of the wet atmosphere in Sm_2_Zr_2_O_7_ is negligible (Figure 5a and Figure 6). In accordance with TG data (Figure 4a), there is no structurally bound water and interstitial protons in disordered Sm_2_Zr_2_O_7_. Sm_2_Zr_2_O_7_ has predominantly intrinsic oxygen-ion conductivity [29,45]. It is known from the literature that Sm_2_Zr_2_O_7_ is an intrinsic ionic conductor and that the fraction of anti-site pairs in its cation sublattice reaches 8.1% [45]. Disordered pyrochlore structures typically contain not only cation anti-site pairs but also oxygen vacancies:

Sm_Sm_^x^ + Zr_Zr_^x^ → Sm_Zr_^′^ + Zr_Sm_^●^ (1)

O_o_^×^ → V_O(48f)_^●●^ + O_i (8b)_^″^ (2)

Therefore, the pyrochlore structure of Sm_2_Zr_2_O_7_ contains a sufficient concentration of intrinsic oxygen vacancies, but only a small part of them can be involved in the formation of mobile protons at sufficient hydrophilicity of the compound (Figure 6).

With the Ca doping, extrinsic oxygen-ion conductivity appears. Clearly, Ca substitution on the Sm^3+^ site also produces oxygen vacancies, and most of them can take part in proton transfer (Figure 6). In the case of heterovalent substitution of Ca^2+^ for Sm^3+^, the following scheme can be written for the (Sm_2−x_Ca_x_)Zr_2_O_7−x/2_ solid solutions:

4CaO + 4Sm_Sm_^×^+2Zr_Zr_^×^ + 3O_O_^×^ → 4Ca_Sm_**^′^** + 2Sm_Zr_^′^ + 3V_O_^●●^ + Sm_2_Zr_2_O_7_. (3)

Analyzing Figure 6 one can see, that with Ca doping, the bulk conductivity decreases below 600 °C, but at the same time, the effect of the influence of the wet atmosphere increases. Clearly, the extrinsic oxygen vacancies can be involved in the formation of mobile protons at sufficient hydrophilicity of the compound:

H_2_O + V_O_^••^ + O_O_^×^ = 2(OH)_O_^•^. (4)

This process leads to decreasing of the oxygen-ion conductivity contribution across Sm_2−x_Ca_x_Zr_2_O_7−x/2_ (x = 0, 0.05) series (Figure 6). Further decrease in the bulk oxygen-ion conductivity for the Sm_2−x_Ca_x_Zr_2_O_7−x/2_ (x = 0.1) solid solution is associated with a deviation from stoichiometry inside of grains owing to the grain-boundary CaZrO_3_ perovskite-based phase formation. It is possible that intrinsic oxygen vacancies concentration mainly decreases, whereas extrinsic oxygen vacancies number does not change or increases (Figure 6). The authors of Reference [29] also reported that the total electrical conductivity of Sm_2−x_Ca_x_Zr_2_O_7−x/2_ decreases with increasing CaO content below 600 °C in air.

In Reference [46] for (La_1-y_Ca_y_)_2_(Ce_1-x_Zr_x_)_2_O_7-δ_ (y = 0, 0.02, 0.1; x = 0, 0.5, 0.75) pyrochlore–fluorite series was suggested that Ca doping decreases the ionic (oxygen–ion and proton) conductivity owing to the trapping of the mobile ions by the acceptor Ca_La_**^´^**.

In the case of acceptor substitution of Ca for Dy in ordered pyrochlore Dy_2_Ti_2_O_7_, Rietveld refinement of the structure of the (Dy_1.8_Ca_0.2_)Ti_2_O_6.9_ pyrochlore solid solution detected neither cation antistructure pairs nor related oxygen vacancies [47]. All of the oxygen vacancies presented were the result of substitution. A different situation occurs in the case of Sm_1.95_Ca_0.05_Zr_2_O_6.975_. According to the XRD data in Table S1, Sm_1.95_Ca_0.05_Zr_2_O_6.975_ contains not only oxygen vacancies due to Ca substitution for Sm but also vacancies due to cation anti-site pairs (~2–3%). We suppose that degrees of substitution ~5% (x = 0.1) may cause deviations from stoichiometry in the grain bulk and the formation of a small amount of a new perovskite-based phase, according to XRD data (Figure 1, scan 3; the lines (100) and (110) of CaZrO_3_ perovskite are marked by asterisks), that confirms earlier results [29] and correlates well with a small difference in the lattice parameters for Sm_1.95_Ca_0.05_Zr_2_O_6.975_ and Sm_1.9_Ca_0.1_Zr_2_O_6.95_ (Table 2). The formation of the SrHfO_3_ and SrZrO_3_ perovskites as impurity phases was observed upon 10% (x = 0.2) Sr doping on the Gd site in gadolinium hafnate and gadolinium zirconate [28,34]. It seems likely that this is possible in La_1.95_Sr_0.05_Zr_2_O_6.975_ as well [18]. At high synthesis temperatures (higher than 1700 °C) Huo et al. [18] also observed deviations from stoichiometry in the grain bulk of La_1.95_Sr_0.05_Zr_2_O_6.975_.

It is seen in Figure 7a,b that grain boundaries in the Sm_2−x_Ca_x_Zr_2_O_7−x/2_ (x = 0.05, 0.1) pyrochlore solid solutions also have proton conductivity. Grain-boundary conductivity in wet air exceeds that in dry air. In addition, Figure 7a,b illustrates the relationship between bulk and grain-boundary conductivities in dry and wet air. It is seen that, in this case, the total conductivity is determined by the bulk component in all of the samples except Sm_2−x_Ca_x_Zr_2_O_7−x/2_ (x = 0.1) (Figure 7b). In Sm_2−x_Ca_x_Zr_2_O_7−x/2_ (x = 0.1), the total conductivity is limited by grain-boundary conductivity below 650 °C and by bulk conductivity in the range of 650–750 °C. In contrast to Sr-doped gadolinium zirconates and hafnates [28,34] and Sr-doped lanthanum zirconate [18], where the total conductivity is limited by grain-boundary conductivity, an opposite situation occurs for Sm_2−x_Ca_x_Zr_2_O_7−x/2_ (x = 0.05). In both dry and wet air, the grain boundaries in the Sm_2−x_Ca_x_Zr_2_O_7−x/2_ (x = 0.05) solid solution (3 × 10^−3^ S/cm at 600 °C) have a factor of 5–10 higher conductivity in comparison with the grain bulk (7.5 × 10^−4^ S/cm at 600 °C) (Figure 7). The grain-boundary conductivity of the Sm_2−x_Ca_x_Zr_2_O_7−x/2_ (x = 0.1) solid solution (5% Ca substitution) is slightly lower than that of Sm_2−x_Ca_x_Zr_2_O_7−x/2_ (x = 0.05) (Figure 7). The difference between the bulk and grain-boundary conductivity contributions decreases with increasing substitution degree in Sm_2−x_Ca_x_Zr_2_O_7−x/2_ (x = 0.05, 0.1), which is obviously due to an increase in structural disorder inside of Sm_2−x_Ca_x_Zr_2_O_7−x/2_ (x = 0.1) grains owing to the grain-boundary CaZrO_3_ perovskite-based phase formation.

The total conductivity of the Sm_2−x_Ca_x_Zr_2_O_7−x/2_ (x = 0, 0.05, 0.1) series in dry and wet air is presented in Figure 8. It is seen that, in both dry and wet air, the total conductivity decreases with increasing Ca content below 600 °C.

Perovskite CaZrO_3_ exists in two polymorphs: orthorhombic (at low temperatures) and cubic (at high temperatures), with a transition between them near 1950 °C [48]. The conductivity of unsubstituted perovskite CaZrO_3_, whose typical lines are present in the XRD pattern of Sm_2−x_Ca_x_Zr_2_O_7-d_ (x = 0.1) (Figure 1, scan 3; the lines of the CaZrO_3_ impurity phase are marked by asterisks), is markedly lower: ~6 × 10^−5^ S/cm at 600 °C [49]. Ca_1-x_ZrO_3-δ_ (0 ≤ x ≤ 1) ceramics with cation nonstoichiometry have mixed proton–hole conductivity [49], which decreases with increasing cation nonstoichiometry. At the same time, there is a widely known pioneering study of the proton conductivity of rare-earth-doped calcium, strontium, and barium zirconates with the perovskite structure [50]. Iwahara et al. [50] failed to obtain high-density ceramics for electrochemical measurements in the case of CaZrO_3_-based solid solutions doped with Y, Nd, Dy, and Yb on the Zr site. Such ceramics were produced only for Al, Ga, In, and Sc dopants and had proton conductivity above 1.3 × 10^−4^ S/cm at 600 °C. In a recent study [51], glycine–glycerin–nitrate combustion synthesis followed by annealing at 1500 °C for 5 h made it possible to obtain dense (98%) CaZr_0.95_Sc_0.05_O_3-δ_ ceramics with an orthorhombically distorted pyrochlore structure (sp. gr. *Pnma*) and 600 °C proton conductivity as high as 6 × 10^−4^ S/cm. Therefore, the proton conductivity of CaZrO_3_ doped with rare earths, including Sm and Gd, can be rather high, suggesting that proton conductivity can contribute to the grain-boundary conductivity of the synthesized Sm_2−x_Ca_x_Zr_2_O_7−x/2_ (x = 0.05, 0.1) pyrochlore solid solutions. In this context, there is considerable interest in a study by Davies et al. [52], who analyzed the proton conductivity of CaZrO_3_ doped with large (La and Nd) and small (Yb and Sc) rare-earth cations using EXAFS and computer simulation. The highest proton conductivity was found in CaZrO_3_ doped with the small cations on the Zr site: CaZr_0.95_R_0.05_O_3-δ_ (R = Yb, Sc). In this study, Sm and Gd—intermediate rare-earth cations—can act as dopants on the Zr site.

As mentioned above, undoped CaZrO_3_ undergoes an orthorhombic (sp. gr. *Pnma*)–cubic (sp. gr. *Pm3m*) polymorphic transformation at 1950 °C [48]. It is reasonable to expect high proton conductivity of the Sm doped CaZrO_3_ perovskite phase (~ 4 × 10^−3^ S/cm at 600 °C (Figure 7)) [35] forming on grain boundaries of pyrochlore Sm_2_Zr_2_O_7_ based phase used as substrates for the growth of high-conductivity cubic perovskite Sm doped CaZrO_3_ phase at 1600 °C.

### 3.5. Conductivity of the Gd_2−x_Ca_x_Zr_2_O_7−x/2_ (x = 0.05, 0.1) Solid Solutions in Dry and Wet Air

**Figure 9:**
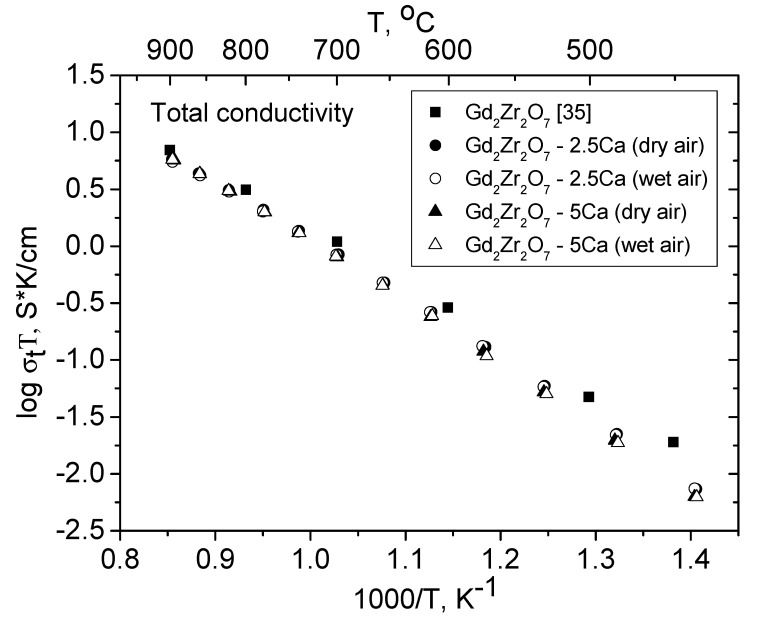
The total conductivity of Gd_2−x_Ca_x_Zr_2_O_7−x/2_ (x = 0.05, 0.1) in dry and wet air. The bulk conductivity data for undoped Gd_2_Zr_2_O_7_ in ambient air are borrowed from Moreno et al. [35].

We failed to separately assess the grain-boundary conductivity of the Gd_1.95_Ca_0.05_Zr_2_O_6.975_ solid solution, so Figure 9 and Table 4 present the activation energy for the total conductivity of Gd_2_Zr_2_O_7_ (it was synthesized using mechanical activation and annealing at 1500 °C for 36 h and its conductivity was measured in ambient air [35]), Gd_1.95_Ca_0.05_Zr_2_O_6.975_ and Gd_1.9_Ca_0.1_Zr_2_O_6.95_ in dry and wet air (this work). It is seen that Ca doping has a negligible effect on the Arrhenius plot and that the Gd_2−x_Ca_x_Zr_2_O_7−x/2_ (x = 0.05, 0.1) solid solutions have no proton conductivity. In a previous study, Fournier et al. [26], who synthesized a Gd_2−x_Ca_x_Zr_2_O_7−x/2_ (x = 0, 0.05, 0.2, 0.3) series at 1700 °C, observed a marked reduction in the total conductivity with increasing Ca content in air, and, most likely, this is due to the transition from pyrochlore structure to fluorite. Unfortunately, structural studies in Reference [26] were not conducted. In the present work, the synthesis temperature was below (1600 ° C) and throughout the Gd_2−x_Ca_x_Zr_2_O_7−x/2_ (x = 0, 0.05, 0.1) series (Figure 9), the total conductivity at 600 °C was actually the same and is ~ (3–5) × 10^−4^ S/cm.

Recall that Gd_2_Zr_2_O_7_ is the most disordered pyrochlore oxide in the rare-earth zirconate family. It is probably for this reason that Ca doping of highly disordered pyrochlore solid solutions has no advantageous effect, especially at such high temperatures of synthesis as 1600–1700 °C, since under these conditions not pyrochlores, but fluorites are formed. Therefore, the conductivity of pyrochlore Sm_2−x_Ca_x_Zr_2_O_7−x/2_ (x = 0.05) is slightly higher: 8 × 10^−4^ S/cm at 600 °C (Figure 6), than that of fluorite Gd_2−x_Ca_x_Zr_2_O_7−x/2_ (x = 0.05).

**Figure 10:**
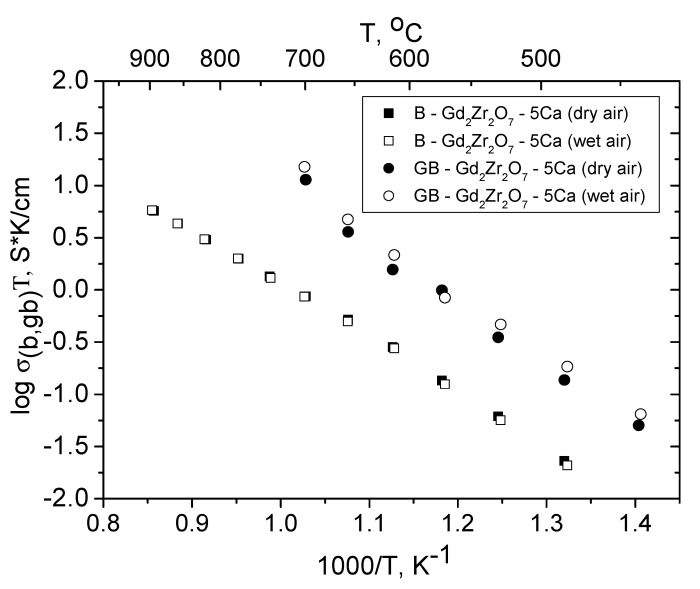
Bulk and grain-boundary conductivities of Gd_2−x_Ca_x_Zr_2_O_7−x/2_ (x = 0.1) in dry and wet air.

Figure 10 compares the bulk and grain-boundary conductivities of the Gd_2−x_Ca_x_Zr_2_O_7-d_ (x = 0.1) solid solution, for which we were able to separately assess these components. It is seen that, like in the Sm series, there is grain-boundary proton conductivity (below 700 °C) and that the grain-boundary conductivity is an order of magnitude higher than the bulk conductivity in a wide temperature range: 440–700 °C. According to Figure 9, there is no bulk proton conductivity in the Gd series, but the Gd_2−x_Ca_x_Zr_2_O_7−x/2_ (x = 0.1) solid solution has grain-boundary proton conductivity (Figure 10). The loss of bulk proton conductivity in Gd_2−x_Ca_x_Zr_2_O_7−x / 2_ (x = 0.05) can be associated with a high degree of disordering of its pyrochlore structure. If we calculate Gd_2−x_Ca_x_Zr_2_O_7−x/2_ (x = 0.05) as disordered pyrochlore, we obtain that Gd_2−x_Ca_x_Zr_2_O_7−x/2_ (x = 0.05) contains 36–50% anti-site pair (Table 2 and Table S2) and is actually fluorite. The loss of the preferred directions for bulk proton transfer is associated with a strong disordering of the pyrochlore structure, despite hydrophilic properties of Gd_2−x_Ca_x_Zr_2_O_7−x/2_ (x = 0.05, 0.1) pyrochlores (Figure 4c). The slight weight loss (Figure 4c) found in TG experiments for Gd_2−x_Ca_x_Zr_2_O_7−x/2_ (x = 0.1) is most likely related to the proton component of the Gd-doped CaZrO_3-δ_ perovskite.

As in the case of the Sm_2−x_Ca_x_Zr_2_O_7−x/2_ (x = 0.1) solid solution, we assume that a high-conductivity of Gd-doped phase is due to cubic perovskite CaZrO_3_, which could be present on grain boundaries of Gd_2−x_Ca_x_Zr_2_O_7−x/2_ (x = 0.1), with conductivity a factor of 2.5 higher than that of Sm doped CaZrO_3_ (Figs.10 and 7 b). Recently the admixture of CaZrO_3_ was observed for the same composition Gd_1.9_Ca_0.1_Zr_2_O_6.95_, obtained by a hydrothermal process followed by annealing at 1500 °C for 4 h [38]. Note that, unlike SrZrO_3_ and SrHfO_3_ [28,34], the intergranular Gd-doped CaZrO_3_ phase has higher conductivity (both the oxygen-ion and proton components) and does not limit the total conductivity of the material.

### 3.6. Relationship between the Grain Boundary and Bulk Conductivities of Ordered and Disordered Pyrochlores

Perriot et al. [53] performed molecular dynamics simulations to investigate the role of grain boundaries (GBs) on ionic diffusion in pyrochlores as a function of the GBs type, Ln/M ratio and level of cation disorder in Ln_2_M_2_O_7_ pyrochlores. They reported that in highly disordered pyrochlores, the diffusive behavior at the GBs is bulk-like, and the two contributions (bulk and GB) can no longer be distinguished.

Analysis of the present data for disordered pyrochlore Gd_2−x_Ca_x_Zr_2_O_7−x/2_ (x = 0.05) (Table 2, Figure 1a) from this point of view also suggests that there is no distinction between the bulk and grain-boundary conductivities of this material (not shown here). Distinctions emerge at a higher degree of substitution, in Gd_2−x_Ca_x_Zr_2_O_7−x/2_ with *x* = 0.1 (Figure 10), due to the presence of an intergranular impurity phase with the perovskite structure. This is also evidenced by the calculation results in Table 2. Analysis of the Rietveld refinement results for disordered Gd_2−x_Ca_x_Zr_2_O_7−x/2_ (x = 0.05) indicates that this solid solution actually has the fluorite structure (Table 2). Then the bulk and grain-boundary conductivities of Gd_2−x_Ca_x_Zr_2_O_7−x/2_ (x = 0.1) can readily be distinguished (Figure 10) because of the formation of an intergranular CaZrO_3_ -based perovskite phase. In the opposite case, the bulk and grain-boundary conductivities would be the same for both compositions Gd_2−x_Ca_x_Zr_2_O_7−x/2_ (x = 0.05, 0.1).

In the case of the more ordered pyrochlore zirconate Sm_2−x_Ca_x_Zr_2_O_7−x/2_ (x = 0.05), the bulk and grain-boundary conductivities can readily be distinguished and the latter is markedly higher (Figure 7a,b). The calculation results in Table S1 indicate the presence of ~ 2–3 % cation anti–site pairs (Sm_Zr_^′^ + Zr_Sm_^●^) in Sm_2−x_Ca_x_Zr_2_O_7−x/2_ (x = 0.05), along with Ca substitution on the Sm site. With Ca doping increasing, the disorder in Sm_2−x_Ca_x_Zr_2_O_7−x/2_ (x = 0.1) pyrochlore increases due to the deviation of the stoichiometry of the grain interior because formation of the intergranular Sm doped CaZrO_3_ phase. As a result, the difference between the bulk and grain-boundary components decreases (Figure 7a,b).

If bulk conductivity decreases and grain-boundary conductivity rises with dopant concentration in the samples obtained at high temperature T ≥ 1600 °C, this is most likely due to the formation of a new, intergranular phase, accompanied by deviations from stoichiometry in the grain bulk. The likely reason for this effect is that the degree of substitution exceeds the solubility limit in the pyrochlore phase with a given Ln/M (M = Ti, Zr, Hf, Sn) ratio. If both bulk and grain-boundary conductivities increase with dopant concentration, the dopant is most likely evenly distributed between the grain bulk and grain boundaries of the ordered pyrochlore phase, as in the case of Ca-, Mg-, and Zn-doped Ln_2_Ti_2_O_7_ (Ln = Dy, Ho, Yb) [54].

Raising the Ca concentration to above the optimal one at a given temperature leads to deviations from stoichiometry in the grain bulk and second-phase (perovskite) precipitation on grain boundaries. Heating of doped pyrochlores also leads to deviations from stoichiometry in the grain bulk and second-phase precipitation on grain boundaries. In the case of doping of pyrochlores with a lower degree of cation disorder (less than 4.5% antistructure pairs; e.g., Yb_2_Ti_2_O_7_ [55]), an effective dopant of suitable size is evenly distributed between the grain bulk and grain boundaries, increasing the oxygen vacancy concentration both in the grain bulk and on grain boundaries.

It is interesting to note that in Reference [29], where the synthesis of the Sm_2−x_Ca_x_Zr_2_O_7−x/2_ series was carried out at a higher temperature of 1700 °C, the degree of substitution of Ca for Sm in the Sm_2−x_Ca_x_Zr_2_O_7−x/2_ was x = 0.025, and in this paper, where the synthesis was carried out at 1600 °C, the degree of substitution is 0.05 ≤ x ≤ 0.1.

### 3.7. Oxygen–Ion Conductivity of the Sm_2−x_Ca_x_Zr_2_O_7−x/2_ (x = 0.05, 0.1), Gd_2−x_Ca_x_Zr_2_O_7−x/2_ (x = 0.1) Solid Solutions in the 700 ≤ T≤ 950 °C Temperature Interval

**Figure 11:**
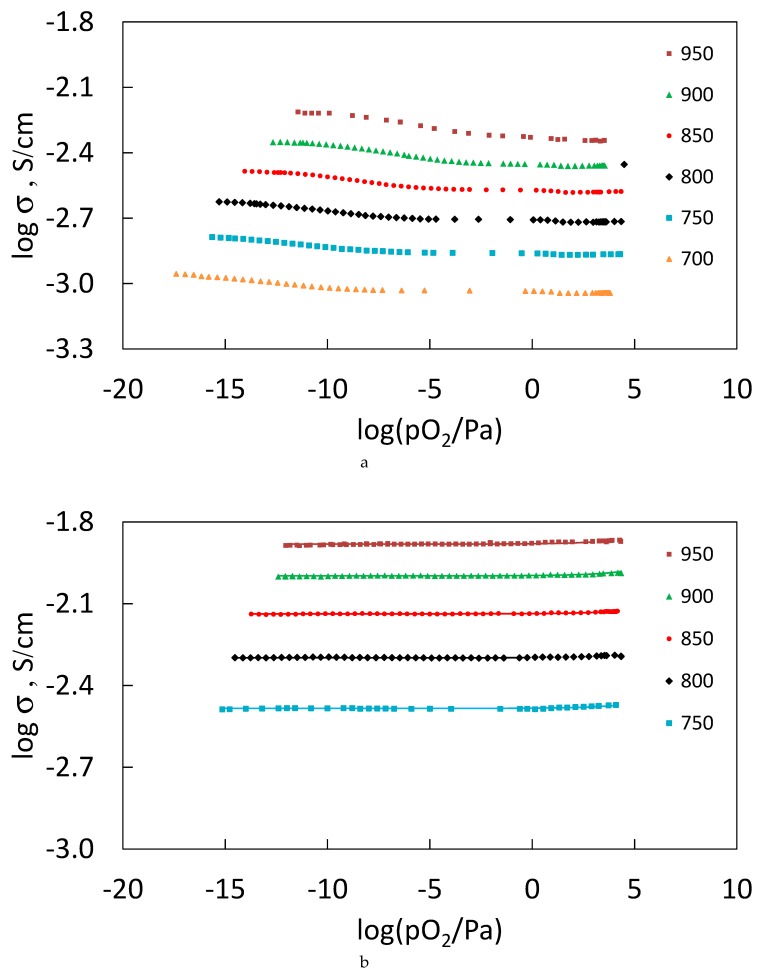

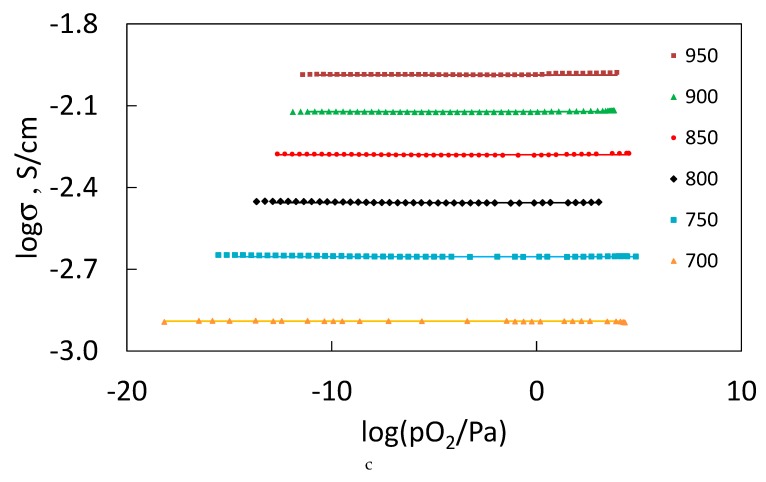
Total electrical conductivity of (**a**) Sm_2−x_Ca_x_Zr_2_O_7−x/2_ (x = 0.05), (**b**) Sm_2−x_Ca_x_Zr_2_O_7−x/2_ (x = 0.1), (**c**) Gd_2−x_Ca_x_Zr_2_O_7−x/2_ (x = 0.1) as a function of the oxygen partial pressure, for temperatures between 700 and 950 °C.

The total conductivity measurements as a function of oxygen partial pressure of Sm_2−x_Ca_x_Zr_2_O_7−x/2_ (x = 0.05, 0.1) (Figure 11a,b) and Gd_2−x_Ca_x_Zr_2_O_7−x/2_ (x = 0.1) (Figure 11c), shows a typical ionic conductivity plateau, which should be attributed to the intrinsic and extrinsic oxygen vacancies co-existed in the structure.

For the Ca-doped Sm_2_Zr_2_O_7_ pyrochlore the increase of Ca content enhances the ionic conductivity as expected due to the positive oxygen vacancies formation the compensate for the addition of the negative defect Ca_Sm_**^´^**. For the higher Ca content composition (x = 0.1) the oxygen partial pressure dependences suggest that the extrinsic behavior dominates, while for the composition with lower Ca content (x = 0.05), a slight increase of the conductivity was observed at low oxygen partial pressures, which suggests that the intrinsic n-type conductivity can start to contribute to the overall conductivity for this working conditions. It can be assumed that, at 0.05 < x < 0.1, the maximum of calcium doping of the gadolinium sublattice is reached, as a result of which optimal values of ionic conductivity are obtained. However, at x = 0.1, a proton-conducting phase with a perovskite structure is formed at the grain boundaries.

For the disordered Ca-doped Gd_2_Zr_2_O_7_ solid solution with fluorite structure, the extrinsic behavior was also founded for x = 0.1 composition, however with a slight lower conductivity than the correspondent composition with x = 0.1 for the Ca- doped Sm_2_Zr_2_O_7_. Structural differences and the possible occurrence of second phases in both systems can justify these differences in conductivity.

The possibility of having n-type conductivity for high oxygen partial pressures is not evident, and there are also no changes in the activation energy values between high and low oxygen pressures, suggesting the same conductivity mechanism in all oxygen partial pressure ranges. In this temperature range, due to differences in activation energies, the overall conductivity is controlled by the bulk resistance, so possible changes in the grain-boundary conductivity mechanism are not observed. It should be noted that in this temperature range the impedance spectra only show the arc corresponding to the electrode/interface behavior and it is only possible to determine the total conductivity, which occurs in the left intersection of this arc with the x-axis.

## 4. Conclusions

Doping with divalent Ca cations increases the conductivity of the rare-earth zirconates and titanates only if they have the pyrochlore structure, and the effect is larger for ordered Ln_2_Zr_2_O_7_ (Ln = La) and Ln_2_Ti_2_O_7_ (Ln = Dy – Yb) pyrochlores [55]. In the case of highly disordered pyrochlores near the pyrochlore–fluorite morphotropic phase boundary, there is no such effect. In undoped Sm_2_Zr_2_O_7_, the concentration of intrinsic oxygen vacancies responsible for proton transport much lower than that in Sm_2−x_Ca_x_Zr_2_O_7−d_ (x = 0.05, 0.1) Ca-doped solid solutions. Extrinsic oxygen vacancies, as a result of Ca-doping process, to a greater degree than that of intrinsic vacancies can be involved in the formation of mobile protons.

The 500 °C proton conductivity contribution of Sm_2−x_Ca_x_Zr_2_O_7−x/2_ (x = 0.05, 0.1) is ~ 1 × 10^–4^ S/cm. Sm_2−x_Ca_x_Zr_2_O_7−x/2_ (x = 0.05, 0.1) solid solutions have proton conductivity both in the grain bulk and on grain boundaries, in agreement with TG data, and below 600 °C their bulk oxygen–ion and proton conductivity decreases with increasing Ca-doping level, but at the same time, the effect of the influence of the wet atmosphere on conductivity increases. It is possible that Ca doping decreases the ionic (oxygen-ion and proton) conductivity owing to the decreasing of unit cell volume because the change of Ca coordination number from 8 to 7 in the pyrochlore structure exists at low temperatures or it is possible due to the negative effect of deviation from stoichiometry inside of grain bulk of Sm_2−x_Ca_x_Zr_2_O_7−x/2_ (x = 0.1).

The Ca-doping effect exists in Sm_2−x_Ca_x_Zr_2_O_7−x/2_ (x = 0. 0.05, 0.1) solid solutions above 700 °C, where they are pure oxygen-ion conductors: the bulk conductivity of the Ca-doped solid solutions exceeds that of undoped Sm_2_Zr_2_O_7_ and the region of dominant oxygen conductivity increases with increasing dopant concentration.

The highly disordered Gd_2−x_Ca_x_Zr_2_O_7−x/2_ (x = 0.1) pyrochlore has oxygen-ion bulk conductivity, whereas proton transport contributes to its grain-boundary conductivity. The loss of bulk proton conductivity in Gd_2−x_Ca_x_Zr_2_O_7−x/2_ (x = 0.05, 0.1) can be associated with formation of the fluorite structure. Ca-doping of highly disordered pyrochlores (containing ~8% or more anti-site pairs [44,45]) usually reduces their bulk conductivity.

In both series, grain-boundary conductivity exceeds bulk conductivity in the temperature range up to 700–750 °C. In the case of Gd_2−x_Ca_x_Zr_2_O_7−x/2_ (x = 0.1), they differ by an order of magnitude. As a result of the pyrochlore-to-fluorite morphotropic phase transition, bulk proton conductivity disappears and oxygen-ion conductivity decreases. The high grain-boundary proton conductivity of Ln_2−x_Ca_x_Zr_2_O_7−x/2_ (Ln = Sm, Gd; x = 0.05, 0.1) is attributable to the formation of a CaZrO_3_-based impurity phase doped with Sm or Gd, respectively, the composition of which is assumed to be CaZr_1−x_Ln_x_O_3−δ_ (Ln = Sm, Gd).

We believe that, in the synthesis of Ca- and Sr-doped rare-earth zirconates with the pyrochlore structure, the ceramics preparation temperature plays an important role. After high-temperature synthesis near 1600 °C and above, there is typically a grain-boundary contribution to conductivity and, accordingly, the bulk and grain-boundary contributions can be separated. Grain-boundary conductivity often limits the total conductivity, but may have an advantageous effect, exceeding bulk conductivity. As a result, the process is only limited by bulk conductivity. In most cases, grain-boundary conduction in divalently doped pyrochlore zirconates after annealing at ~1600 °C and higher temperatures is due to deviations from stoichiometry in the grain bulk and the formation of MZrO_3_–based (M = Ca, Sr) impurity phases, which increase or decrease grain-boundary conductivity.

Another factor favourable for this process (grain-boundary conduction appearance) is the ratio of the ionic radii of the host and dopant cations in the pyrochlore structure. For example, Ca is too large dopant for the Ln_2_Zr_2_O_7_ (Ln = Sm, Gd) pyrochlores and, according to the present results, the Ln_2−x_Ca_x_Zr_2_O_7−x/2_ (Ln = Sm, Gd; x = 0.1) materials (doped with just 5% Ca) contain a proton-conducting intergranular impurity phase.

The results of the study of bulk proton conductivity contribution in Sm_2_Zr_2_O_7_ and Gd_2−x_Ca_x_Zr_2_O_7−x/2_ (x = 0.1) with disordered pyrochlore and fluorite structure, respectively, show that the bulk proton conductivity in them is insignificant or absent.

The optimal value of Ca doping for proton conductivity in Sm_2−x_Ca_x_Zr_2_O_7−x/2_ (x = 0, 0.05, 0.1) is found at 0.05 < x < 0.1, which corresponds to theoretical calculations x = 0.08 [30].

To sum up, this work presents the oxygen-ion/proton and bulk/gb conductivity ratios for the Sm_2−x_Ca_x_Zr_2_O_7−x/2_ (x = 0, 0.05, 0.1) pyrochlores and Gd_2−x_Ca_x_Zr_2_O_7−x/2_ (x = 0.05, 0.1) fluorites, shows the relationship between high values of conductivity (oxygen–ion and proton) of 3+/4+ pyrochlores and location of 3+/4+ pyrochlores near morphotropic boundaries, analyzes optimal temperature annealing and optimal dopant concentration for different rare-earth zirconates as potential materials for proton-conducting fuel cells (PC-SOFCs).

## Figures and Tables

**Table 1:** Characteristics of the compounds under investigation.

Sample No.	Formula	Sintering Annealing	Phase Composition According to XRD	Color	Relative Density, %
1	Sm_2_Zr_2_O_7_	1600 °C, 10 h	Pyrochlore (P)	Cream	89
2	Sm_1.95_Ca_0.05_Zr_2_O_6.975_	1600 °C, 4 h	Pyrochlore (P)	Cream	91.6
3	Sm_1.9_Ca_0.1_Zr_2_O_6.95_	1600 °C, 4 h	Pyrochlore (P)	Reddish-brown	92.6
4	Gd_1.95_Ca_0.05_Zr_2_O_6.975_	1600 °C, 4 h	Fluorite (F)	Gray	89.1
5	Gd_1.9_Ca_0.1_Zr_2_O_6.95_	1600 °C, 4 h	Fluorite (F)	Gray	89

**Table 2:** Rietveld data for Sm_2−x_Ca_x_Zr_2_O_7−x/2_ (x = 0, 0.05, 0.1) and Gd_2−x_Ca_x_Zr_2_O_7−x/2_ (x = 0.05, 0.1).

Composition	Site	Occupancy	x	y	z	*R*_exp_, % *R*_wp_, % *R*_p_, % GOF	Parameter *a,* Å
Sm_2_Zr_2_O_7_ space group: $Fd\bar{3}m$	Sm_Sm_ (16d)	0.905(5)	0.500	0.500	0.500	3.46 4.16 3.28 1.20	*a* =10.5975(1)
	Zr_Sm_ (16d)	0.095(5)	0.500	0.500	0.500		
	Zr_Zr_ (16c)	0.905(5)	0.000	0.000	0.000		
	Sm_Zr_ (16c)	0.095(5)	0.000	0.000	0.000		
	O(1) (8b)	1	0.375	0.375	0.375		
	O(2) (48f)	1	0.339	0.125	0.125		
Sm_1.95_Ca_0.05_Zr_2_O_6.975_ space group: $Fd\bar{3}m$	Sm_Sm_ (16d)	0.975(1)	0.500	0.500	0.500	3.36 4.49 3.47 1.34	*a* = 10.5925(1)
	Zr_Sm_ (16d)	0	0.500	0.500	0.500		
	Ca_Sm_ (16d)	0.025	0.500	0.500	0.500		
	Zr_Zr_ (16c)	1	0.000	0.000	0.000		
	Sm_Zr_ (16c)	0	0.000	0.000	0.000		
	Ca_Zr_ (16c)	0	0.000	0.000	0.000		
	O(1) (8b)	1	0.375	0.375	0.375		
	O(2) (48f)	1	0.339	0.125	0.125		
Sm_1.9_Ca_0.1_Zr_2_O_6.95_ space group: $Fd\bar{3}m$	Sm_Sm_ (16d)	0.930(2)	0.500	0.500	0.500	3.51 4.94 3.79 1.41	*a* = 10.5923(1)
	Zr_Sm_ (16d)	0.030(2)	0.500	0.500	0.500		
	Ca_Sm_ (16d)	0.04	0.500	0.500	0.500		
	Zr_Zr_ (16c)	0.960(2)	0.000	0.000	0.000		
	Sm_Zr_ (16c)	0.030(2)	0.000	0.000	0.000		
	Ca_Zr_ (16c)	0.01	0.000	0.000	0.000		
	O(1) (8b)	1	0.375	0.375	0.375		
	O(2) (48f)	1	0.339	0.125	0.125		
Gd_1.95_Ca_0.05_Zr_2_O_6.975_ space group: $Fd\bar{3}m$	Gd_Gd_ (16d)	0.585(40)	0.500	0.500	0.500	2.92 3.72 2.88 1.27	*a* = 10.5326(1)
	Zr_Gd_ (16d)	0.400(40)	0.500	0.500	0.500		
	Ca_Gd_ (16d)	0.015	0.500	0.500	0.500		
	Zr_Zr_ (16c)	0.600(40)	0.000	0.000	0.000		
	Gd_Zr_ (16c)	0.390(40)	0.000	0.000	0.000		
	Ca_Zr_ (16c)	0.01	0.000	0.000	0.000		
	O(1) (8b)	1	0.375	0.375	0.375		
	O(2) (48f)	1	0.339	0.125	0.125		
Gd_1.95_Ca_0.05_Zr_2_O_6.975_ space group: $Fm\bar{3}m$	Gd_Gd_ (4a)	0.4875(1)	0.000	0.000	0.000	2.92 3.67 2.82 1.26	*a =* 5.2663(1)
	Zr_Gd_ (4a)	0.5000(1)	0.000	0.000	0.000		
	Ca_Gd_ (4a)	0.0125(1)	0.000	0.000	0.000		
	O(1) (8c)	0.8750	0.250	0.250	0.250		
Gd_1.9_Ca_0.1_Zr_2_O_6.95_ space group: $Fd\bar{3}m$	Gd_Gd_ (16d)	0.580(40)	0.500	0.500	0.500	2.80 3.66 2.83 1.31	*a* = 10.5321(1)
	Zr_Gd_ (16d)	0.390(40)	0.500	0.500	0.500		
	Ca_Gd_ (16d)	0.03	0.500	0.500	0.500		
	Zr_Zr_ (16c)	0.610(40)	0.000	0.000	0.000		
	Gd_Zr_ (16c)	0.370(40)	0.000	0.000	0.000		
	Ca_Zr_ (16c)	0.02	0.000	0.000	0.000		
	O(1) (8b)	1	0.375	0.375	0.375		
	O(2) (48f)	1	0.339	0.125	0.125		
Gd_1.9_Ca_0.1_Zr_2_O_6.95_ space group: $Fm\bar{3}m$	Gd_Gd_ (4a)	0.475(1)	0.000	0.000	0.000	2.80	*a* = 5.2661(1)
	Zr_Gd_ (4a)	0.500(1)	0.000	0.000	0.000	3.62	
	Ca_Gd_ (4a)	0.025(1)	0.000	0.000	0.000	2.81	
	O(1) (8c)	0.875	0.250	0.250	0.250	1.29	

**Table 3:** Comparison of the nominal cations stoichiometry and SEM/EDX point analysis of Sm_1.9_Ca_0.1_Zr_2_O_6.95_ ceramics.

Analyzing Area	Concentration, at.%			Ratio		
	Sm	Ca	Zr	[Zr]/[Ca]	[Sm]/[Ca]	[Zr]/[Sm]
Nominal stoichiometry	47.5	2.5	50.0	20.0	19.0	1.1
**SEM/EDX point analysis**						
Micron-size grain(s) only (well co-sintered)	55.2	1.7	43.1	26.5	33.9	0.8
	51.5	2.5	46.0	18.5	20.7	0.9
Grain boundary	57.5	1.6	40.9	25.7	36.1	0.7
	57.6	1.6	40.8	25.5	36.0	0.7
	51.2	2.0	46.8	22.9	25.1	0.9
100–300 nm grains on the surface of micron-size well co-sintered grains	57.5	2.0	40.5	20.7	29.3	0.7
	51.8	3.1	45.1	14.4	16.6	0.9
Individual grains with the size of 0.6–1.1 mkm	73.6	2.9	23.5	8.1	25.2	0.3
	49.1	4.9	46.0	9.4	10.1	0.9
	58.0	3.7	38.3	10.3	15.6	0.7
Micron-size agglomerate formed from small grains, Figure 3c	26.0	54.5	19.5	0.4	0.5	0.7
	20.3	66.7	13.0	0.2	0.3	0.6

**Table 4:** Activation energy for total, bulk and gb conductivity in ambient air in the compounds studied.

Compound	Structure (XRD Data)	Temperature, °С	Activation Energy for Total Conductivity, eV (Dry Air)	Activation Energy for Total Conductivity, eV (Wet Air)
Sm_2_Zr_2_O_7_	Pyrochlore	Above 500 °С	0.7	0.7
		Below 500 °С	0.83	0.73
Sm_1.95_Ca_0.05_Zr_2_O_6.975_	Pyrochlore	300–900 °С	0.82	0.79
Sm_1.9_Ca_0.1_Zr_2_O_6.975_	Pyrochlore	300–900 °С	0.95	0.89
Gd_1.95_Ca_0.05_Zr_2_O_6.975_	Fluorite	Above 580 °С	0.99	0.99
		Below 580 °С	1.15	1.15
Gd_1.9_Ca_0.1_Zr_2_O_6.95_	Fluorite	Above 580 °С	1.03	1.03
		Below 580 °С	1.17	1.17

## Supplementary Materials

The following are available online at https://www.mdpi.com/1996-1944/12/15/2452/s1, Table S1. Comparison of Rietveld refinement factors for Sm_1.95_Ca_0.05_Zr_2_O_6.975_ composition, Table S2. Comparison of Rietveld refinement factors for Sm_1.9_Ca_0.1_Zr_2_O_6.95_ composition, Figure S1. Mapping of the area with the micron-size agglomerate formed from small grains (Figure 3c): (a) Ca; (b) Zr; (c) Sm; (d) overlapping of the maps for individual elements.
