# Supplementary material for: Evolution of Oxygen–Ion and Proton Conductivity in Ca-Doped Ln2Zr2O7 (Ln = Sm, Gd), Located Near Pyrochlore–Fluorite Phase Boundary
Source: Materials (Basel). 2019 Aug 1;12(15):2452. doi: 10.3390/ma12152452 (PMC6696096; doi:10.3390/ma12152452)
Supplement: Supplementary file 1 [file materials-12-02452-s001.pdf]

materials

MDPI

Supplementary Materials

Evolution of Oxygen–Ion and Proton Conductivity in Ca-Doped $Ln_2Zr_2O_7$ (Ln = Sm, Gd), Located Near Pyrochlore–Fluorite Phase Boundary

A.V. Shlyakhtina [1,*], J.C.C. Abrantes [2], E. Gomes [2], N.V. Lyskov [3], E.Yu. Konysheva [4,5], S.A. Chernyak [6], E.P. Kharitonova [6], O.K. Karyagina [7], I.V. Kolbanev [1], L.G. Shcherbakova [1]

Table S1. Comparison of Rietveld refinement factors for $Sm_{1.95}Ca_{0.05}Zr_2O_{6.975}$ composition.

A crystallographic position			B crystallographic position			Parameter *a*, Å	Refinement factors
Sm_{Sm} (16d)	Zr_{Sm} (16d)	Ca_{Sm} (16d)	Zr_{Zr} (16c)	Sm_{Zr} (16c)	Ca_{Zr} (16c)		
0.975	0	0.025	1	0	0	10.5925(1)	R_{exp}= 3.36%, R_{wp} = 4.49%, R_p=3.47%, GOF=1.34
0.965	0.01	0.025	0.99	0.01	0	10.5925(1)	R_{exp}=3.36%, R_{wp} = 4.49%, R_p=3.47%, GOF=1.33
0.955	0.02	0.025	0.98	0.02	0	10.5925(1)	R_{exp}= 3.36%, R_{wp} = 4.49%, R_p=3.49%, GOF=1.34
0.955	0.03	0.015	0.97	0.02	0.01	10.5925(1)	R_{exp}= 3.36%, R_{wp} = 4.49%, R_p=3.47%, GOF=1.33
0.965	0.015	0.02	0.985	0.01	0.05	10.5925(1)	R_{exp}= 3.36%, R_{wp} = 4.50%, R_p=3.48%, GOF=1.34

Table S2. Comparison of Rietveld refinement factors for $Sm_{1.9}Ca_{0.1}Zr_2O_{6.95}$ composition.

A crystallographic position			B crystallographic position			Parameter *a*, Å	Refinement factors
Sm_{Sm} (16d)	Zr_{Sm} (16d)	Ca_{Sm} (16d)	Zr_{Zr} (16c)	Sm_{Zr} (16c)	Ca_{Zr} (16c)		
0.92	0.03	0.05	0.97	0.03	0	10.5923(1)	R_{exp} =3.51%, R_{wp} = 4.94%, R_p= 3.79%, GOF=1.41
0.91	0.04	0.05	0.96	0.04	0	10.5923(1)	R_{exp} = 3.51%, R_{wp} = 4.95%, R_p=3.79%, GOF=1.41
0.89	0.06	0.05	0.94	0.06	0	10.5923(1)	R_{exp} = 3.51%, R_{wp} = 4.95%, R_p= 3.79%, GOF=1.41
0.93	0.03	0.04	0.96	0.03	0.01	10.5923(1)	R_{exp} = 3.51%, R_{wp} = 4.94%, R_p= 3.79%, GOF=1.41
0.935	0.03	0.035	0.955	0.03	0.015	10.5923(1)	R_{exp} =3.51%, R_{wp} = 4.95%, R_p=3.79%, GOF=1.41
0.9	0.08	0.02	0.89	0.08	0.03	10.5923(1)	R_{exp} =3.51%, R_{wp} = 4.95%, R_p=3.79%, GOF=1.41

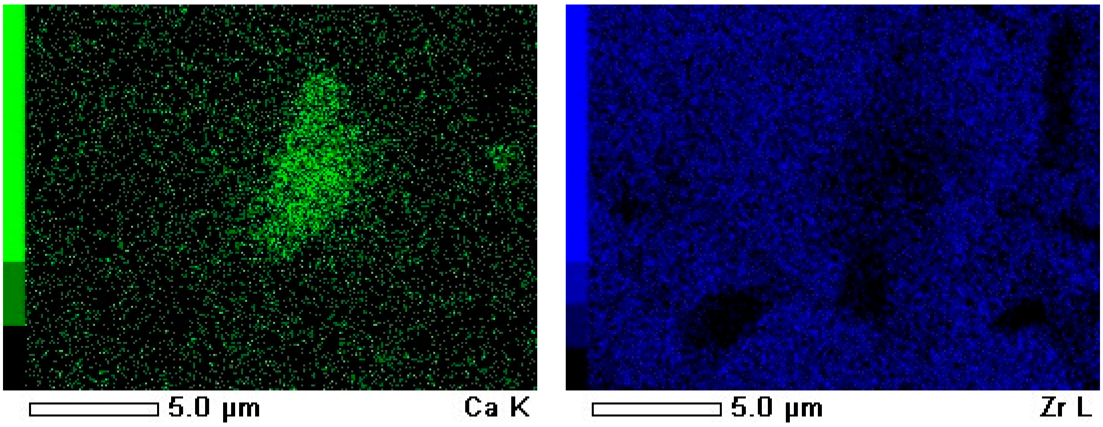

(**a**) (**b**)

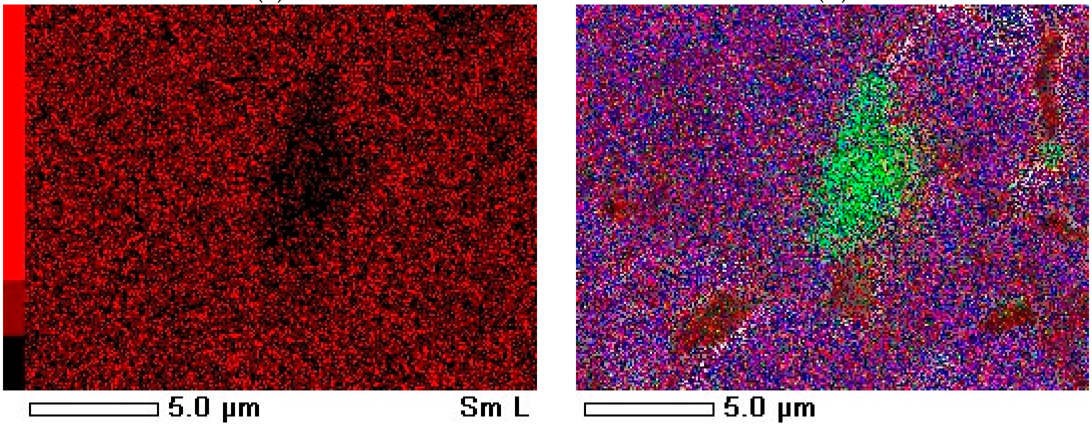

(**c**) (**d**)

Figure S1. Mapping of the area with the micron-size agglomerate formed from small grains (Figure 3c): (a) Ca; (b) Zr; (c) Sm; (d) overlapping of the maps for individual elements.
